# Cerebral microvascular endothelial cell-derived extracellular vesicles regulate blood − brain barrier function

**DOI:** 10.1186/s12987-023-00504-6

**Published:** 2023-12-19

**Authors:** Baharak Hosseinkhani, Gayel Duran, Cindy Hoeks, Doryssa Hermans, Melissa Schepers, Paulien Baeten, Joren Poelmans, Britt Coenen, Kübra Bekar, Isabel Pintelon, Jean-Pierre Timmermans, Tim Vanmierlo, Luc Michiels, Niels Hellings, Bieke Broux

**Affiliations:** 1 University MS Center, Campus Diepenbeek, Diepenbeek, Belgium; 2 Neuro-Immune Connections and Repair Lab, Department of Immunology and Infection, Biomedical Research Institute, UHasselt, Diepenbeek, Belgium; 3 Laboratory of Angiogenesis and Vascular Metabolism, Center for Cancer Biology (CCB), VIB, KU Leuven, Leuven, Belgium; 4 Department of Oncology, Leuven Cancer Institute (LKI), KU Leuven, Leuven, Belgium; 5 Department Psychiatry and Neuropsychology, School for Mental Health and Neuroscience, Maastricht University, Maastricht, The Netherlands; 6 Department of Neuroscience, Biomedical Research Institute, Faculty of Medicine and Life Sciences, Hasselt University, Hasselt, Belgium; 7 Laboratory of Cell Biology & Histology/Antwerp Centre for Advanced Microscopy (ACAM), University of Antwerp, Universiteitsplein 1, Antwerp, 2610 Belgium; 8 Bionanotechnology group, Biomedical Research Institute, UHasselt, Diepenbeek, Belgium; 9 Universiteit Hasselt, Martelarenlaan 42, Hasselt, Belgium

**Keywords:** Multiple sclerosis, BBB, EV

## Abstract

Autoreactive T lymphocytes crossing the blood-brain barrier (BBB) into the central nervous system (CNS) play a crucial role in the initiation of demyelination and neurodegeneration in multiple sclerosis (MS). Recently, extracellular vesicles (EV) secreted by BBB endothelial cells (BBB-EC) have emerged as a unique form of cell-to-cell communication that contributes to cerebrovascular dysfunction. However, the precise impact of different size-based subpopulations of BBB-EC-derived EV (BBB-EV) on the early stages of MS remains unclear. Therefore, our objective was to investigate the content and function of distinct BBB-EV subpopulations in regulating BBB integrity and their role in T cell transendothelial migration, both in vitro and in vivo. Our study reveals that BBB-ECs release two distinct size based EV populations, namely small EV (sEV; 30-150 nm) and large EV (lEV; 150-300 nm), with a significantly higher secretion of sEV during inflammation. Notably, the expression patterns of cytokines and adhesion markers differ significantly between these BBB-EV subsets, indicating specific functional differences in the regulation of T cell migration. Through in vitro experiments, we demonstrate that lEV, which predominantly reflect their cellular source, play a major role in BBB integrity loss and the enhanced migration of pro-inflammatory Th1 and Th17.1 cells. Conversely, sEV appear to protect BBB function by inducing an anti-inflammatory phenotype in BBB-EC. These findings align with our in vivo data, where the administration of sEV to mice with experimental autoimmune encephalomyelitis (EAE) results in lower disease severity compared to the administration of lEV, which exacerbates disease symptoms. In conclusion, our study highlights the distinct and opposing effects of BBB-EV subpopulations on the BBB, both in vitro and in vivo. These findings underscore the need for further investigation into the diagnostic and therapeutic potential of BBB-EV in the context of MS.

**Supplementary Information:**

The online version contains supplementary material available at 10.1186/s12987-023-00504-6.

## Background

Multiple sclerosis (MS) is a multifocal inflammatory and degenerative autoimmune disease ranked as the prime cause of neurological disability in young and middle-aged adults worldwide [1, 2]. Demyelination of nerves in the central nervous system (CNS) causes a variety of symptoms such as fatigue, vision problems, cognitive decline and motor deficits [3]. At the root of its neuropathology, during initiation of disease, there is a pervasive state of inflammatory interactions between two major cellular players, namely immune cells and blood-brain barrier endothelial cells (BBB-EC) [4]. The healthy and normal brain vasculature contains tightly sealed EC exhibiting low pinocytotic activity. This, together with the expression of tight junction (TJ) and adherens junction (AJ) proteins, strictly regulates movements of peripheral cells, ions, and molecules into the CNS, thereby maintaining brain homeostasis [5–7]. Upon chronic inflammation in MS, pro-inflammatory cytokines such as IFN-γ, IL-17, and IL-1β released by immune cells mediate the breaching of the BBB by upregulation of adhesion molecules (ICAM-1, VCAM-1, E-selectin, and PECAM-1), and loss of junctional integrity [8]. Upregulation of adhesion molecules leads to an increased influx of peripherally activated immune cells, mostly CD8^+^ and CD4^+^ T cells, over the BBB at the post-capillary venules where they cause a perpetuation of the local immune response in the CNS, resulting in neurological disabilities [9–12].

Cell-cell communication is critical for immune responses, and chemokines and cytokines have been considered the main facilitators of the communication [13]. Over the past few years, the discovery of extracellular vesicles (EV) has revolutionized the understanding of cell–cell communication in the development of several diseases [14, 15]. In 2018, the International Society for Extracellular Vesicles (ISEV) published a systematic review of guidelines, namely MISEV2018, for the classification, characterization and functional assessment of EV. MISEV2018 has highlighted that EV subpopulations can be purified based on their size, density and surface markers [16]. Size-based EV subpopulations have been commonly classified as large EV (lEV) (> 200 nm) or small EV (sEV) (< 200 nm), which are isolated using a low sedimentation speed (< 20,000 g) or ultra-centrifugal sedimentation speed (> 100,000 g), respectively. Cell-derived EV can carry a wide variety of molecules (lipids, proteins, m(i)RNAs) that facilitate intercellular communication [17, 18]. All immune cells are able to secrete EV, and these EV have been shown to actively engage in the immune responses [19]. Several studies have demonstrated that the cargo of EV is a mirror of the physiological condition of the cell of origin, and that EV can serve as potential biomarkers of disease [20].

The small size of EV allows entrance over protective barriers in the body, such as the BBB. Here it can secrete its cargo inside the CNS and potentially perpetuate immune responses [21, 22]. An elegant review from Palacio et al. [23] described what is known so far on the interaction of the BBB and EV. In short, it has been demonstrated that EV released by BBB-EC contribute to impaired barrier function of the BBB leading to increased immune cell infiltration in the CNS [24, 25], a key hallmark of MS. An elevated level of CD31-positive microvesicles (currently identified as lEV) has been reported in the blood and cerebrospinal fluid (CSF) of MS patients [26]. Even though the number of studies into EV in MS has quadrupled in the last years, only a limited number of studies have been devoted to investigating the biological role of EV in disease progression, and these studies focused particularly on the early stages of MS [14, 27–29]. In vitro, it was found that EV upregulate ICAM-1 and VCAM-1 on BBB-EC, leading to increased T cell adhesion after exposure to EV [25, 30]. However, the precise immunomodulatory contents of different subpopulations of EV derived from BBB-EC and their mechanisms of action at the inflamed BBB site, both in vitro and in vivo, have yet to be fully elucidated. Given the unique ability of EV to transport inflammatory proteins and RNA, we hypothesized that size-based subpopulations of BBB-EV may contain functional inflammatory modulators capable of inducing inflammation-associated markers in neighboring healthy cells, thereby contributing to the pathology of multiple sclerosis (MS).

To test this hypothesis, we collected three distinct EV subpopulations based on their sedimentation speeds at two centrifugal forces (2,000 and 10,000 × g; EV-2 K and EV-10 K) and one ultra-centrifugal force (110,000 × g; EV-100 K) from the culture supernatant of human BBB-EC (hCMEC/D3). The BBB-EC were either untreated (uEV) or treated with TNF-α and IFN-γ to induce inflammatory stress (tEV). Following the MISEV2018 guidelines, we characterized the EV fractions using transmission electron microscopy (TEM), nanoparticle tracking analysis (NTA), and western blotting (WB) to examine their morphology, size distribution, quantities, and EV-specific markers. Our results demonstrated that inflammatory stress triggered the release of two distinct size-based populations of EV from BBB-EC, with a higher tendency towards the secretion of EV-100 K. Additionally, we found that inflammatory BBB-EV were enriched with key chemoattractant cytokines involved in T cell migration. Using in vitro migration assays with human-derived cells, we examined the impact of sEV (EV-100 K) and lEV (EV-2 K and EV-10 K) on breaching BBB-EC and on the migration of T cells across a BBB-EC monolayer. Furthermore, we investigated the association between sEV and lEV and the progression of a preclinical model of MS using the experimental autoimmune encephalomyelitis (EAE) mouse model. Notably, treatment with sEV in vivo resulted in reduced clinical symptoms in the EAE model, while lEV treatment exacerbated the disease. To assess whether different EV subpopulations (EV-2 K, EV-10 K and EV-100 K) actively contribute to BBB dysfunction, we investigated the upregulation of adhesion molecules, inflammatory markers, and loss of barrier properties. These findings highlight the opposing roles of sEV and lEV in BBB disruption within the context of neuroinflammation.

## Materials and methods

### Study subjects

Peripheral blood samples were collected from healthy controls in collaboration with the University Biobank Limburg (UBiLim, Hasselt, Belgium). In total, 5 donors were used (1 male and 4 female) with ages ranging between 23 and 44 years (mean ± SEM: 33.8 ± 4.3). This study was approved by the local ethical committee and informed consent was obtained from all donors.

### Cell culture

Human cerebral microvascular endothelial cells (hCMEC/D3) were provided by Tebubio (le Perray-en-Yevelines, France) and were seeded at 1-1.2 × 10^6^ cells/cm^2^ onto pre-coated plates or inserts with 75 µg/mL rat tail collagen type I solution (Merck) between passages 27 and 34. Cells were grown in EGM™-2MV Microvascular Endothelial Cell Growth Medium-2 BulletKit™ (CC-3202, Lonza) supplemented with 2.5% fetal bovine serum (FBS) (Gibco™, Thermo Fisher Scientific) up to 80–90% confluency to minimize apoptosis as recommended by MISEV [16]. Characteristics and stability of the used cell line are extensively described [31]. For the production of EV, confluent cells were rinsed twice with PBS (Lonza) and a mixture of rhTNF-α and rhIFN-γ (Peprotech, Londen, UK) at a final concentration of 10 ng/mL were added in refreshed medium supplemented with 2.5% exosome-depleted fetal bovine serum (EXO-FBS-250 A-1, System Bioscience) for 24 h or left untreated in exosome-depleted medium. Exposure time and concentration of inflammatory cytokines were as previously described in Hermans et al. [32]. To prevent the induction of apoptosis in cells, and thus the presence of apoptotic bodies in EV preparation steps, cell culture supernatants were collected after 24 h from approximately 6–8 × 10^6^ cells/mL (from ~ 80–90% confluency) with 98% viability. For expression and functional studies, medium was changed to experimental medium: EBM™-2 Basal Medium (CC-3156, Lonza) supplemented with 5 ng/ml human fibroblast growth factor (hFGF), 1.4 µM hydrocortisone, 10 mg/ml gentamicin, 1 mg/ml amphotericin (A2942, all Merck) and 2.5% FCS after reaching 80% confluency at least 24 h before starting treatment. During treatment, cells were placed on serum-reduced (0.25% FCS) experimental medium (as described above) without hydrocortisone. All culturing took place in a humidified atmosphere condition of 37 °C/5% CO2, on rat tail collagen type I coated plastics (75 µg/ml) and routine mycoplasma contamination tests were performed throughout the culturing.

### Isolation of size-based EV populations

Isolation of sEV and lEV was performed on pooled cell culture supernatants of approximately 24 million cells using a differential (ultra)centrifugation method. Although most studies in the field of EV research apply differential centrifugation for the enrichment of large-sized and small-sized EV fractions derived from different cell types at g-forces of 10,000–20,000 x g (10-20 K) and > 100,000 x g (100 K), respectively [15, 35], ISEV has recently suggested a new classification system for EV based on centrifugation conditions. Here, EV were isolated according to their proposal where EV that sediment at 100,000 × g are categorized as sEV, as opposed to exosomes. EV that sediment at speeds lower than 20,000 × g are classified as medium EV (mEV), which can include microvesicles and ectosomes. Lastly, EV that sediment at 2,000 × g are designated as lEV, representing large fragments of cells or large apoptotic bodies [33, 34]. To avoid cross contamination with apoptotic bodies in the lEV fraction, cells were grown up to 80–90% confluency as mentioned previously. Briefly, 40 ml of supernatant was pooled and centrifuged at 300 ×g for 10 min at 4 °C to remove cell debris. To collect lEV (EV-2 K and EV-10 K), supernatants were first transferred to new tubes and centrifuged in a S-4-72 fixed angle rotor (Eppendorf- VWR) at 2000 ×g for 20 min at 4 °C to obtain the EV-2 K pellet. A second centrifugation step was done on 2 K-free supernatant for 40 min at 10,000 ×g at 4°Cs to obtain the EV-10 K pellet. To pellet the sEV (EV-100 K), the 10 K-free supernatant was ultra-centrifuged in a Ti-70 rotor (L-90 Beckman centrifuge, Fullerton, CA, USA) at 100,000 ×g for 3 h at 4 °C. Depending on the downstream analysis, pellets were suspended in either 1 mL of exosome-depleted medium, Pierce RIPA (89,900, Thermo Fisher Scientific, MA, USA) or extraction buffer (ab193970, Abcam Ltd., Cambridge, UK) and stored at − 20 °C for short term storage or -80 °C for long-term storage.

EV for functional assays were further purified using Sepharose CL-2B (17-0140-01, VWR) size exclusion chromatography (SEC) as described by [35]. 1 mL pooled EV-enriched fractions (F4 and F5) were then upconcentrated using Amicon-Ultra 0.5 Centrifugal Filter Units with 10 kDa cutoff (UFC501096, Merck). In addition, to prove the role of EV in functional assays, EV were depleted from EV-2 K, EV-10 K and EV-100 K fractions using Exosome Isolation Pan Kit: EV (130-110-912, Miltenyi Biotec) following the manufacturer’s protocol.

### Transmission electron microscopy (TEM)

The protocol for TEM-imaging of EV has been previously described [35]. Briefly, droplets of the sample were placed on clean Parafilm and a Nickel TEM grid was placed on top of the droplets for 60 min. The grids with adherent EVs were fixed with 2% glutaraldehyde for 10 min, washed 5 times and transferred to 2% uranyl acetate for 15 min. The grids were then incubated in 0.13% methyl cellulose and 0.4% uranyl acetate for 10 min and dried at room temperature before examination with Tecnai G2 Spirit BioTWIN (FEI, Eindhoven, The Netherlands). Images were taken at 120 kV.

### Nanoparticle tracking analysis (NTA)

The mean size, concentration and size distribution of EV were quantified using the NanoSight NS300 system (Malvern Ltd, Sysmex Belgium N.V.) equipped with a 532 nm laser. EV suspensions were diluted with PBS over a range of concentrations to obtain between 10 and 100 particles per frame. The camera level was set to 14. Samples were injected into the sample chamber and measured five times for 60 s with a syringe pump speed of 75 µl/s. Acquisitions were captured and analyzed using NTA software 3.2 (NanoSight. Malvern Ltd) and the threshold was set to 9. Five measurements were taken for each sample (at least nine biological replicates), mean and standard deviation were calculated and plotted using GraphPad Prism 9 software (GraphPad Software, San Diego, CA, USA). EV concentration was reported as particles/mL and EV size (nm) in mean values.

### Fluorescence labeling of EV and their uptake

The membrane and RNAs loaded in EV were fluorescently labelled by adding five microliters of Vybrant™ DiD (Thermofisher, Belgium) and SYTO™ RNASelect™ (Thermofisher, Belgium) to one ml of isolated EV and incubated at room temperature for two hours. Free dyes were removed from labeled EV using Sepharose CL-2B (17-0140-01, VWR) SEC as described previously. Pooled EV fractions (F4 and F5) were concentrated using Amicon-Ultra 0.5 Centrifugal Filter Units with 10 kDa cutoff (UFC501096, Merck) at 4 °C.

To compare the uptake of different sized based fractions of EV, hCMEC/D3 cells were grown in pre-coated eight-well culture plates, for 24 h before EV incubation. 10^9^ labeled EV were added to cells and incubated for 24 h. After incubation, cells were washed with PBS and fixed with PFA 2%. Nuclear staining was performed with 4’,6-diamidino-2-phenylindole (DAPI) at a final concentration of 10 µg/ml for 30 min at 37 °C. Following the staining, images were captured using a Leica DM2000 LED (Leica Microsystems, Heidelberg, Germany) attached to a digital camera and Leica Application Suite X (LAS X) software (Leica Microsystems).

### Protein extraction and quantification

Protein content of cells and EV lysates in either RIPA (89,900, Thermo Fisher Scientific, MA, USA) supplemented with Protease Inhibitor Cocktail (P8340, Sigma-Aldrich, MO, USA) or extraction buffers (ab193970, Abcam) was determined using Pierce BCATM protein assay reagent Kit (Thermo Fisher Scientific, MA, USA) and micro Pierce BCATM protein assay reagent kit (Thermo Fisher Scientific, MA, USA), respectively. The procedure was done following the manufacturer’s specifications. Optical densities of standards and samples were read at OD595 nm using a Multiskan™ FC microplate absorbance reader (Thermo Fisher Scientific, MA, USA).

### Western blotting

**Fig. 1:**
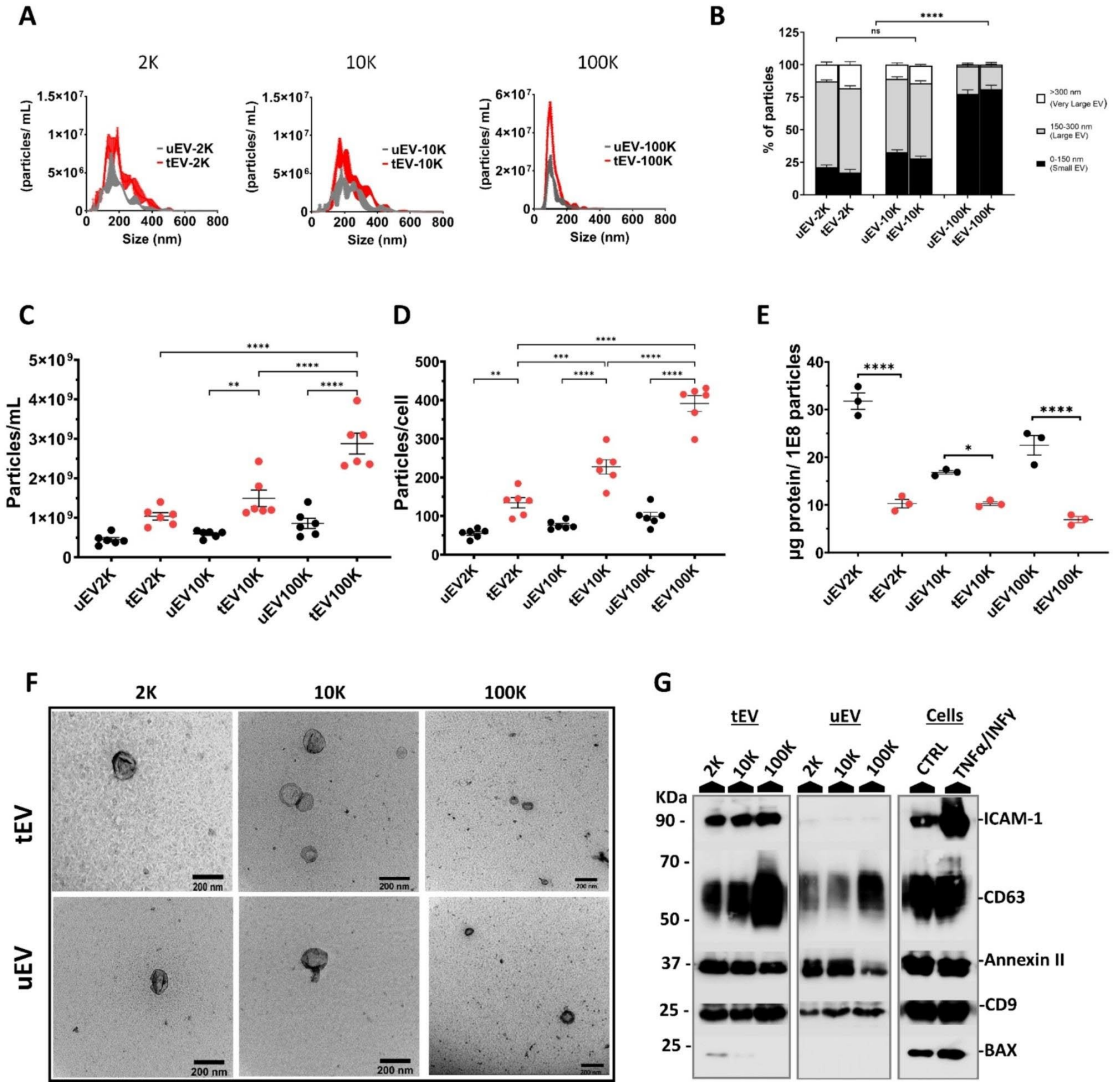
Characterization of size-based subpopulations of EV released from TNFα/IFNγ treated (tEV) and untreated hCMEC/D3 endothelial cells (uEV). (**A**) EV size distribution profile (nm) and concentration (particles/ml) of both uEV (black) and tEV (red) fractionated based on their size at low (2,000×g = EV-2 K and 10,000 ×g = EV-10 K) and ultra-centrifugal force (100,000 ×g = EV-100 K) using NTA. Histograms represent the mean ± SD of nine independent biological experiments (n = 9). (**B**) The percentage of small EV (0- 150 nm), large EV (150–300 nm) and very large EV (> 300 nm) in the 2 K, 10 and 100 K fractions. Data were obtained from nine independent biological experiments (n = 9). (**C**–**D**) Data represent (**C**) the particle number per milliliter of uEV (black) and tEV (red) of size based EV subpopulations (n = 6). (**D**) Total amount of protein per particle uEV (black) and tEV (red) size based EV subpopulations (n = 3). Data of NTA analysis are presented as means ± SEM (n = independent biological experiments, 1 symbol per batch) and one-way ANOVA Tukey’s multiple comparison was used to determine significance between multiple groups: ns, no significance, *p < 0.05 **P < 0.01, ****P < 0.0001. (E) TEM images of uEV and tEV in different recovered sized based fractions (2 K, 10 K, and 100 K) (Scale bar = 200 nm). (F) Representative western blots of CD9 (24 kDa), CD63 (30–70 kDa) as classical EV membrane-bound markers, ICAM-1 (85–110 kDa) as inflammation-associated marker, Annexin II (38 kDa) as a cytosolic marker and a proapoptotic -associated protein (BAX ~ 25 kDa) as a negative control in EV lysate side-by-side with the lysate of producing untreated (CTRL) and inflamed (TNFα/IFNγ) cells. Five micrograms of EV proteins were loaded on the gels. The same trends were detected in western blot data of at least three independent samples (n = 3)

EV and cell pellets were lysed in RIPA buffer (89,900, Thermo Fisher, MA, USA) containing a protease inhibitor cocktail (P8340, Sigma-Aldrich, MO, USA) and denatured at 95 °C for five minutes. EV pellets were normalized based on total proteins in 10^8^ EV (Fig. 1D) before loading in western blot gel. For cell pellets, 5 µg of protein was loaded. Both were first separated by SDS-PAGE with either 12% or 4–7,5% polyacrylamide gels at 80 V for 30 min, followed by 140 V for 45–60 min. The proteins were then transferred onto a polyvinylidene fluoride membrane (Immobilon R, Merck Millipore Ltd) for minimum 1 h at 350 mA. The membranes were blocked in 5% Powdered Milk/PBS or 5% Powdered Milk /TBS 0.1% Tween (TBS-T) for two hours and incubated overnight at 4 °C with primary antibodies: mouse monoclonal anti-human ICAM-1 (1:1000; clone 15.2, sc-107, Santa Cruz Biotechnology), Annexin II (1:1000; clone C-10, sc-28,385 Santa Cruz Biotechnology), CD63 (1:1000; clone Ts63, Thermo Fisher Scientific), CD9 (1:1000; clone Ts9, Life Technologies), ZO-1 (1:1000, 61-7300, Invitrogen), rabbit anti-BAX antibody (1:500; clone E63, ab32503, Abcam), and Claudin-5 (1:1000; 34-1600, Life Technologies) in 5% Powdered Milk /PBS or 5% Powdered Milk /TBS-T. Next, the secondary antibodies (either rabbit anti-mouse HRP-conjugated or goat anti-rabbit HRP-conjugated; both from DAKO) at 1:1000 dilution were added to the membrane for 1 h at room temperature after three times washing with PBS-T. The blots were developed using Western Bright ^TM^ Sirius (K-12,043-C20, Advansta, CA, USA) for the EV lysate or Western Bright ^TM^ Quantum (K-12,042- C20, Advansta, CA, USA) for the cell lysate samples. The corresponding bands were detected by the ImagerQuant™TL (Amersham Imager 680, GE Healthcare) detection system. Band intensities were determined by quantifying the mean pixel gray values using ImageJ software. Western blot analysis for EV markers was performed on visual differences only as no documented housekeeping proteins are reported to normalize EV protein content [36, 37].

### RNA isolation, cDNA synthesis, and quantitative real-time PCR

For quantitative polymerase chain reaction (qPCR) of BBB-EC treated with different fractions of size-based EV, hCMEC/D3 were grown as described in pre-coated 6 well plates at 5 x 10^5^ cells/2 ml. On the day of treatment, cells were washed twice with PBS (Lonza) and treated by adding an equal amount of EV (10^9^ EV/mL) or 10 ng/ mL TNF-α/IFN-γ for 24 h in the refreshed serum-reduced experimental medium. Cells were collected from the plate by scraping in RLT buffer containing 1% β-mercapto-ethanol. Total RNA was isolated from cell pellets using the RNeasy Mini Kit from (Qiagen, Germany) according to the manufacturer’s protocol. RNA concentrations were checked by Nanodrop™ 2000/2000c Spectrophotometer (Thermo Fisher Scientific). Afterwards, complementary DNA (cDNA) was generated from 1 µg of total RNA using qScript cDNA SuperMix (Quanta bioscience, CA, USA) following the manufacturer’s protocol. PCR reactions contained 10 ng cDNA, Fast SYBR Green (Applied Biosystems, 4,385,612), and 10 µM forward and reverse primer mixture (Integrated DNA Technologies, Leuven, Belgium). Quantitative PCR was performed utilizing a StepOnePlus Real-Time PCR detection system (Life technologies) and universal cycle conditions (20s at 95 °C, 40 cycles of 3 s at 95 °C and 30 s at 60 °C). Data as ΔΔCt was normalized to the two most stable housekeeping genes. Primers used in this study are listed in Table 1.

**Table 1:** Primer sequences

Gene	Forward primer (5’ – 3’)	Reverse primer (5’ – 3’)
mTBP*	ATGGTGTGCACAGGAGCCAAG	TCATAGCTACTGAACTGCTG
mYWHAZ*	GCAACGATGTACTGTCTCTTTTGG	GTCCACAATTCCTTTCTTGTCATC
mZO-1	GGCATTCCTGCTGGTTACA	AGGACACCAAAGCATGTGAS
mClaudin-5	GCCCTGCTCAGAACAGACTA	GGCAGTTTGGTGCCTACTTC
mIL-1β	GCTGAAAGCTCTCCACCTCA	AGGCCACAGGTATTTTGTCG
mIL-10	CTTTAAGGGTTACCTGGGTTGC	ATTAAAGGCATTCTTCACCTGC
mIL-17	CAGCGATCATCCCTCAAAGC	GCGCCAAGGGAGTTAAAGAC
mCCL2	CAGCAGGTGTCCCAAAGAAG	CATTTGGTTCCGATCCAGGTT
mCXCL10	CTGCCCACGTGTTGAGATCA	TGGTCTTAGATTCCGGATTCAGA
mCD3	AACACTTTCTGGGGCATCCT	CTGTCTAGAGGGCACGTCAA
mCD4	GTTCAGGACAGCGACTTCTG	GAAGGAGAACTCCGCTGACT
mCD8	AGTGAAGGGGACCGGATTG	GGACATTTGCAAACACGCTT
mFoxp3	CAGAGAGGTATTGAGGGTGGG	GCAGAGTCAGGAGAAGTTGC
hTBP*	TATAATCCCAAGCGGTTTGC	GCTGGAAAACCCAACTTCTG
hCYCA*	AGACTGAGTGGTTGGATGGC	TCGAGTTGTCCACAGTCAGC
hIL-1β	GATGAAGTGCTCCTTCCAGG	GCATCTTCCTCAGCTTGTCC
hIL-18	GACTCTTGCGTCAACTTCAAGG	CAGGCTGTCTTTTGTCAACGA
hIL-6	GAGGAGACTTGCCTGGTGAA	GCTCTGGCTTGTTCCTCACT
hICAM-1	AGCTTCGTGTCCTGTATGGC	ACAGTCACTGATTCCCCGAT
hVCAM-1	AATGTTGCCCCCAGAGATACAACCG	GAGCTGCCTGCTCCACAGGA
hE-selectin	GCACTGTGTGCAAGTTCGC	GGCTTTTGGTAGCTTCCGTC
hClaudin-1	TTGACTCCTTGCTGAATCTGAG	TTCTGCACCTCATCGTCTTC
hClaudin-3	CATCACGTCGCAGAACATCT	GAGTCGTACACCTTGCACTG
hClaudin-5	ACATTGTCGTCCGCGAGTTT	ACTTCTGCGACACGGGCA
hVE-Cadherin	AAACACCTCACTTCCCCATC	ACCTTGCCCACATATTCTCC
hPECAM	ATTGCAGTGGTTATCATCGGAGTG	CTCGTTGTTGGAGTTCAGAAGTGG
hZO-1	CCCGAAGGAGTTGAGCAGGAAATC	CCACAGGCTTCAGGAACTTGAGG

*Housekeeping genes

### Human inflammation antibody array

To simultaneously detect 40 inflammation-associated proteins in EV and cell lysates, a membrane based human inflammation antibody C3 array (C-Series – AAH-INF-3 – RayBiotech Norcross-GA) was purchased from RayBiotech (Boechout, Belgium). Assays were performed according to the manufacturers’ instructions in two biological replicates. Briefly, 25 µg of total EV proteins in extraction buffer (Abcam) was subjected to a pre-blocked membrane and incubated overnight at 4 °C with gentle shaking. Afterwards, the membrane was incubated with the primary biotin-conjugated antibody for 2 h, followed by incubation with HRP conjugated streptavidin antibodies for 1 h at room temperature. Finally, the signal intensity of each array was imaged using the ImageQuant™TL detection system. Intensity of each dot was then quantified using ImageJ open source software (National Institutes of Health, USA). The spot densitometry data generated were imported into Microsoft Excel software to calculate normalized Z score, log2 changes. Then, heatmaps of inflammation-related protein expression were generated using GENE-E open source software. STRING analysis was conducted using high confidence (score 0.7). Cluster analysis was conducted using k-means with a value of k = 3.

### Transendothelial electrical resistance

Transendothelial Electrical Resistance (TEER) of hCMEC/D3 cell monolayers was measured in real time to quantify BBB integrity. In short, 8250 cells in 250 µl growth medium were grown to confluency on collagen-coated 16-well RTCA E-Plates (Agilent, Santa Clara, CA, USA), containing interdigitated gold microelectrodes. The monolayers of hCMEC/D3 cells at 100% confluency were treated for a period of 48 h with TNF-α/IFN-γ solution (10 ng/mL, positive control), and increasing dose (from 5 × 10^7^ to 6 × 10^8^ particles/ml) of uEV, and tEV size-based EV subpopulations. Resistance values (in Ω) were collected at multiple frequencies, ranging from 1 Hz to 1000 kHz at five frequencies per decade, by a palmSens 4 impedance analyzer, controlled by PSTrace software (palmSens BV, Houten, The Netherlands). TEER values were analyzed at a frequency of 6309.57 Hz, reflecting intercellular junctions, and data are depicted as Ω x cm², based on the well’s surface area (0.196 cm^2^). Data were normalized to the time point 0 h of each condition individually and resistance curves were generated using GraphPad Prism software.

### Flow cytometry

After 24 h treatment of hCMEC/D3 with equal amounts of EV subpopulations or 10ng/ml TNF-α/IFN-γ (positive control), cells were collected by scraping and stained with titrated amounts of mouse monoclonal anti-human antibodies against ICAM-1 (PE/Dazzle™ 594 anti-human CD54 Antibody, cat 353,117, BioLegend; 1/500), VCAM-1 (APC anti-human CD106 Antibody, cat 305,810, Bio Legend; 1/250), and VE-Cadherin (FITC Mouse anti-human CD144, Clone 55-7H1, BD Bioscience; 1/10) for 15 min at room temperature. Acquisition was performed on a BD LSRFortessa™ cell analyzer (BD Bioscience, New Jersey, US) using the BD FACSDiva software (BD Bioscience). Data were analyzed with FlowJo software (BD Bioscience) version 10.7.1. Our gating strategy excluded doublets (using classical gating strategy), and defined positive gates using fluorescence minus one controls.

### Transwell memory CD4^+^ T cell migration assays

#### CD4^+^ memory selection

All experiments were done using fresh peripheral blood mononuclear cell (PBMC) isolated from whole blood by density gradient centrifugation (Cedarlane lympholyte, Sheffield, UK). Negative selection of CD4^+^ memory T cells from PBMC using magnetic beads was performed according to the manufacturer’s protocol (130-091-893, Miltenyi Biotec, Leiden, The Netherlands). CD4^+^ memory T cells were used for Boyden chamber migration assays directly following isolation.

#### Migration assay with treated hCMEC/D3 cells

For migration assays, hCMEC/D3 were cultured in collagen coated Thincerts (24 well, translucent, 3 μm, Greiner Bio-One, Vilvoorde, Belgium) at a density of 25 × 10^3^ cells/ cm². On days 3 and 5, hCMEC/D3 were replenished with experimental medium. Growth of the monolayer was followed using measurement of TEER with the EVOM2 Epithelial Voltohmmeter (World precision instruments, Hertfordshire, UK) and reached a plateau phase on day 6. Then, hCMEC/D3 were replenished with serum-reduced experimental medium, and either treated with tEV (sEV or lEV), inflammatory cytokines as described previously or left untreated for 24 h. Prior to adding T cells, hCMEC/D3 on the inserts were washed with serum-reduced experimental medium and transferred to a new plate with fresh serum-reduced experimental medium. Memory CD4^+^ T cells (2.97 × 10^5^- 5 × 10^5^ per insert, 3 inserts per condition) were allowed to migrate for 24 h. After migration, T cells from the well (bottom) and from the insert (top) were collected, counted using an automatic cell counter (Moxi – Orflo technologies, cat.: MXZ000, Ketchum, US), and phenotyped with flow cytometric analyses. Cells adhered to the endothelial cells on the insert were not taken along for further analysis, since detachment of the cells would make it impossible to confirm their prior position (on top of the endothelial cells or below the monolayer). Cells were stained with titrated amounts of live dead (eF506, fixable viability dye (FVD), cat. 65-0866-14, Invitrogen), CD4 (APC eFluor 780 anti-human CD4 Antibody, cat. 47-0048-42, Invitrogen), CD45RO (PerCP/Cy5.5 anti-human CD45RO Antibody cat 304,222, BioLegend), CXCR3 (Brilliant Violet 711 anti-human CD183 Antibody, cat. 353,732, BioLegend), CCR4 (PE anti-human CD194 Antibody, cat. 359,411, BioLegend), CCR6 (PE/Cy7 anti-human CD196 Antibody, cat. 353,417, BioLegend) after which they were incubated for 30 min at room temperature. Next, cells were analyzed by flow cytometry as described previously. Flowcytometric gating is based on the presence and or absence of markers CCR6, CXCR3 and CCR4 the gating strategy can be found in Fig. S1. Results are portrayed as a percentage of the total amount of cells found in their corresponding location, describing the changes in subset composition.

### Mice

Animal experiments were performed using 10- to 12-week old female wild-type (WT) C57BL/6 mice obtained from Envigo. Mice were housed in an accredited conventional animal facility under a 12 h light/dark cycle with free access to food and water. All procedures were conducted in accordance with the EU directive 2010/63/EU and with prior approval from the Hasselt University Ethics Committee for Animal Experiments.

### Experimental autoimmune encephalomyelitis (EAE) and peripheral EV administration

EAE was induced in wild-type C57BL/6 mice using Hooke Labs EAE induction kit (EK-2110) according to the manufacturer’s instructions (Hooke Laboratories, Lawrence, MA, USA). Briefly, mice between 10 and 12 weeks of age were subcutaneously injected with myelin oligodendrocyte glycoprotein (MOG)_35−55_ peptide emulsified in complete Freund’s adjuvant (CFA) containing *Mycobacterium tuberculosis.* Directly after immunization, mice were injected intraperitoneally (i.p.) with 40ng/100µl pertussis toxin (PTX). Weights and neurological deficits were recorded daily by a blinded investigator. Neurological deficits were scored using a standard 5-point scale (0: no symptoms; 1: limp tail; 2: weakness of hind legs; 3: complete paralysis of hind legs; 4: complete hind and partial front leg paralysis; 5: death).

For systemic EV administration, cohorts were randomized according to mean disease severity and weight. Mice were intravenously (i.v.) injected in the tail vein with 100 µL of 1 × 10^9^ EV/mL at the onset of clinical EAE. Control mice received injections of 100 µl PBS on the same day as the EV injection (n = 12 per group). For post-mortem analysis after transcardial perfusion with PBS-heparin at the chronic phase of the disease (28 dpi), transversal halves of the spinal cords were snap-frozen using liquid nitrogen for immunohistochemistry analysis. Additionally, inguinal lymph nodes, spleen and remaining halves of spinal cord and brain were isolated and snap-frozen for gene expression analysis. Mice were selected for further analysis based on the mean clinical score of the experimental group (*n* = 4 per group).

For analysis of the effect of EV administration on peripheral immune responses, as well as detection of any xenogeneic response, mice were i.v. injected in the tail vein with 100 µL of 6.5 × 10^9^ EV/mL at the onset of clinical EAE. Control mice received injections of 100 µl PBS on the same day as the EV injection (*n* = 4 per group). For immunophenotyping of peripheral lymphoid organs, transcardial perfusion with PBS-heparin at 21 dpi was performed, and inguinal lymph nodes and spleen were isolated. A single cell suspension was derived by mechanical transfer through a 70 μm cell strainer (Greiner Bio-One, Vilvoorde, Belgium). For flow cytometric analysis, the following antibodies were used: anti-mouse CD45 Alexa Fluor 700, CD3 FITC, CD4 Pacific Blue, CD8a Brilliant Violet 510, CD19 Brilliant Violet 650, CD11b PERCP/Cy5.5, Ly6C Brilliant Violet 785, IL-4 PE, IL-17 PE/Dazzle 594, IFNγ PE-Cy7 (all BioLegend). Viable cells were identified using Zombie NIR Fixable Viability Kit (BioLegend). Prior to surface staining, cells were incubated with 10% rat serum. For intracellular staining, cells were permeabilized using BD Cytofix/Cytoperm Fixation/Permeabilization Kit (BD Biosciences) according to the manufacturer’s instructions. Samples were acquired on a LSRFortessa using FACSDiva software, and data were analyzed using FlowJo software (all BD Biosciences).

### Immunohistochemistry

Murine spinal cord tissue was cut into 10 μm serial sections using the Leica CM3050S cryostat (Leica Microsystems). The sections were post-fixed with frozen acetone for 10 min, followed by three washes with PBS. Sections were then incubated with the Dako protein blocking buffer (DAKO) for 1 h at RT. To determine leakage of blood vessels, sections were incubated with rabbit anti-laminin (1:2000, Abcam) and donkey anti-IgG Alexa 488 (1:800, Thermo Fisher Scientific). Binding of the primary antibody against laminin was visualized using goat-anti-rabbit Alexa 555-conjugated secondary antibody (Life Technologies) and nuclear staining was performed with DAPI. Finally, sections were incubated with 0.3% Sudan Black (Merck) in 70% ethanol to limit autofluorescence. All slides were mounted with Fluoromount-G™ Mounting Medium (Invitrogen™, Thermo Fisher Scientific). Microscopic analysis was performed using Leica DM2000 LED and Leica Application Suite X (LAS X) software (Leica Microsystems, Heidelberg, Germany). All analyses were performed in ImageJ (FIJI). In all experiments, a control staining was performed by omitting the primary antibody.

### RNA isolation

RNA was isolated from snap-frozen CNS and spleen tissue using the RNeasy® Mini Kit (Qiagen, Venlo, The Netherlands) according to the manufacturer’s protocol. cDNA syntheses and qPCR were performed as described previously in this paper.

### Statistical analysis

Data were presented as mean ± Standard error of means (SEM) of at least three independent samples. One-way analysis of variance (ANOVA) with a Dunn’s multiple comparisons test (Kruskal-Wallis test) and Student’s test using the statistical packages GraphPad Prism (version 10) was applied to evaluate the statistical significance between different treatments. Two-tailed tests at value of * *p* < 0.05 and were considered as statistically significant. NS represented as not significant, *p* > 0.05.

### Data availability

We have submitted all relevant data of our experiments to the EV-TRACK knowledgebase (EV-TRACK ID: EV220166) (Van Deun J, et al. EV-TRACK: transparent reporting and centralizing knowledge in extracellular vesicle research. Nature methods. 2017;14(3):228 − 32).

## Results

### Inflammation-triggered human BBB-EC release two distinct size-based subpopulations of EV

To investigate the release pattern of EV from BBB-EC during inflammation, we followed the MISEV2018 guidelines and conducted a comprehensive profiling of size-based EV subpopulations, as routinely performed in our laboratory. We isolated three fractions of EV subsets from the cell culture supernatant of resting (uEV) or TNF-α/IFN-γ stimulated hCMEC/D3 cells (tEV) based on their sizes at two different centrifugal forces (2,000 × g; EV-2 K and 10,000 × g; EV-10 K) and one step of ultra-centrifugal force (100,000 × g; EV-100 K). Using nanoparticle tracking analysis (NTA), we observed a heterogeneous population of vesicles with a broad peak in the size range of lEV (150–300 nm) in the EV-10 K and EV-2 K fractions. The EV-100 K fraction primarily contained particles within the typical size range of sEV (30–150 nm) (Fig. 1A). Both the 2 and 10 K fractions generally contained lEV, while the EV-100 K fraction was dominated by vesicles in the size range of sEV (Fig. 1B). Importantly, no significant changes in the mean size of vesicles were observed in the EV fractions from TNF-α/IFN-γ treated cells compared to untreated cells (Fig. S2A–B).

In response to inflammation, there was a notable increase in the concentration of EV per mL for all EV subtypes, while the protein concentration decreased (Fig. 1C–D). However, there were no significant differences in the concentration and mean size distribution between tEV-2 K and tEV-10 K, whereas both were significantly different from tEV-100 K, indicating that BBB-EC released only two heterogeneous size-based populations (sEV: EV-100 K; and lEV: EV-2 K and EV-10 K) (Fig. 1C). EV derived from inflamed ECs showed a decrease in amount of protein per 10^8^ particles (Fig. 1D). However, the total concentration of protein did not differ between uEV and tEV, except the 10 K fraction where tEV showed an increase in the total protein concentration (Fig. S2B). Transmission electron microscopy (TEM) images further confirmed that EV-2 K and EV-10 K fractions contained large vesicle structures, while the EV-100 K subsets predominantly consisted of cup-shaped smaller EV (Fig. 1E).

To further characterize the EV fractions according to the MISEV2018 guidelines, we validated the presence of classical EV markers (CD63 and CD9), a cytosolic EV marker (Annexin II), a non-EV-associated marker (BAX; a proapoptotic protein), and an inflammation-associated marker (ICAM-1) using western blot analysis. As depicted in Fig. 1F, CD63, CD9, and Annexin II, which are classical EV markers, were detected in all three fractions derived from inflamed endothelial cells, albeit to varying degrees. CD63 and CD9 exhibited higher expression in the tEV-100 K fraction while Annexin II was predominantly associated with tEV-2 K and tEV-10 K. Furthermore, the non-EV-associated marker BAX was absent in both the 10 and 100 K pellets but detectable at a low level in the 2 K pellet of treated cells, suggesting that larger EV fractions may carry various components of the secreting cell. Notably, the inflammation-associated marker ICAM-1 showed higher levels in tEV compared to uEV fractions (Fig. 1F).

In summary, our findings confirm that inflammation triggers the release of two distinct size-based subpopulations of EV from human BBB-EC, with a significantly higher secretion of EV-100 K compared to EV-2 K and EV-10 K.

### Inflammatory BBB-EV are packed with factors that mediate T cell migration

**Fig. 2:**
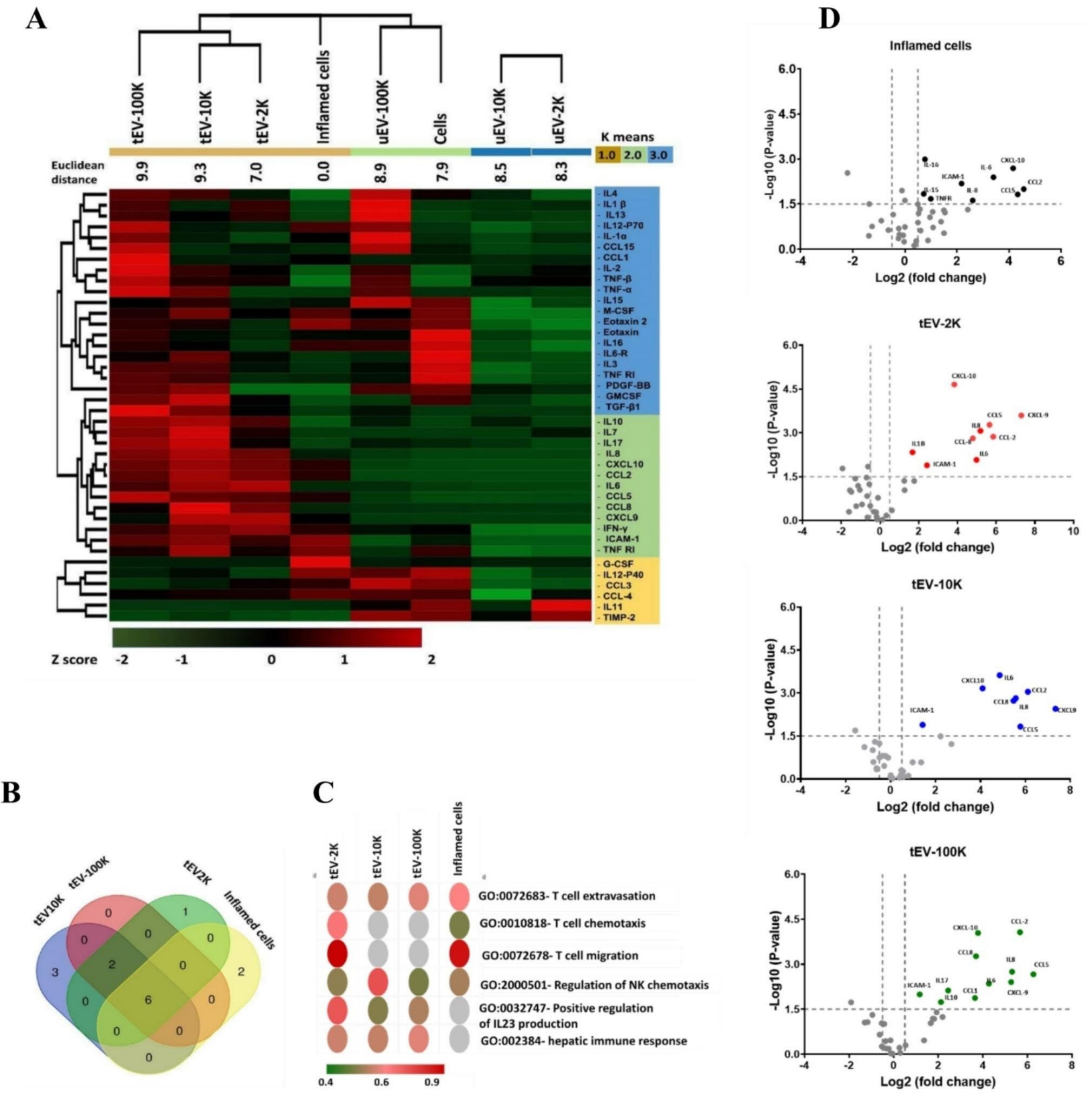
Inflammatory proteome of the size-based EV populations in comparison with their corresponding parental cells (TNFα/IFNγ treated hCMEC/D3). (**A**) Heat map, K-means and Z-score hierarchical clustering of the relative abundance of each inflammatory protein (rows) in size-based fractionated EV, untreated hCMEC/D3 and TNFα/IFNγ treated hCMEC/D3 (columns) using the Euclidean distance metric and a complete linkage method. The colors of the heat map indicate the highly abundant (red) and the basal expression levels (green). Euclidean distance represents the close neighbors to the selected column (TNFα/IFNγ treated hCMEC/D3 (columns)) according to their overall similarity. (**B**) Venn diagram demonstrating the common and distinct differentially expressed inflammatory effectors in the EV of different sizes and TNFα/IFNγ treated hCMEC/D3 (columns). The significance was plotted in gray-red color scale, with gray indicating no significance and red high significance. (**C**) Top five most significantly enriched pathways of differentially expressed inflammatory proteins in the size-based fractionated tEV and corresponding parental cells (TNFα/IFNγ treated hCMEC/D3). Go terms were ranked based on the fold enrichment of -Log 10 (false discovery rate (FDR)) corrected p values. (**D**) Log 2 fold-change vs. Log 10 values of differentially expressed inflammatory proteins in size-based fractionated tEV and corresponding parental cells (TNFα/IFNγ treated hCMEC/D3)

To gain a comprehensive understanding of the cargo carried by EV released from inflamed BBB-EC, we assessed the inflammatory protein content of size-based EV subpopulations (EV-2 K, EV-10 K, and EV-100 K) and compared them with their corresponding parental cells using a membrane-based antibody array. We generated clustered heatmaps and Venn diagrams to visualize the expression patterns of 40 inflammatory markers and compare them between inflamed BBB-EC and their EV derivatives. Hierarchical clustering analysis revealed distinct expression profiles between inflammation-associated cells and tEV, as well as between untreated cells and uEV (Fig. 2A). A total of 31 proteins were expressed, with 14 of them showing differential expression (at least 1.5-fold) in inflamed BBB-EC and tEV compared to untreated cells and uEV. Notably, clustering analysis revealed that tEV-2 K demonstrates the greatest overlap with tEV-10 K this is also seen in the untreated EV fractions.

The Venn diagram (Fig. 2B) showed that 6 out of the 14 differentially expressed proteins (42.8%), namely IL-6, IL-8, ICAM-1, CCL2, CCL5, and CXCL10, were upregulated in both inflamed BBB-EC and their EV derivatives of all sizes compared to untreated cells and uEV. Additionally, among the 14 differentially expressed proteins, 6 proteins (CXCL9, CCL1, CCL8, IL-1β, IL-10, and IL-17) were exclusively differentially expressed in the EV fractions (Fig. 2D and Table S1). Notably, CCL1, IL-10, and IL-17 were exclusively differentially expressed in tEV-100 K (Table S1).

Further gene ontology term enrichment analysis of these proteins revealed their major biological functions, including T cell extravasation (GO:0072683), T cell chemotaxis (GO:0010818), T cell migration (GO:0072678), regulation of natural killer cell chemotaxis (GO:2,000,501), positive regulation of interleukin-23 (IL-23) production (GO:0032747), and hepatic immune response (GO:0002384) (Fig. 2C). These proteins are primarily associated with the positive regulation of IL-23 production, which is a key cytokine involved in Th17 maintenance and expansion. Collectively, these results indicate that the inflammatory proteome of inflamed BBB-EC is predominantly reflected in EV-2 K and EV-10 K, and that BBB-derived EV are enriched with essential chemoattractant cytokines that mediate T cell migration.

In line with our results on size, this content analysis of BBB-EV also indicates that there are only two distinct fractions of EV. Hereafter, migration studies and in vivo experiments, which require high amounts of EV, were performed with pooled EV-2 K and EV-10 K fractions (collectively called lEV) and a separate EV-100 K fraction (sEV).

### Th1 and Th17.1 cell migration through BBB-EC is enhanced by lEV

The migration of circulating leukocytes across the compromised endothelium of the BBB is a critical early step in the development of MS. To investigate the functional implications of intercellular communication mediated by BBB-EV, we examined their impact on T cell migration using an in vitro human BBB model. For this purpose, hCMEC/D3 cells cultured on transwell inserts were treated with different tEV fractions or IFN-y/TNF-α as a positive control. Isolated memory CD4^+^ T cells were used to mimic physiological conditions of MS disease, where autoreactive memory T cells migrate across the BBB, and are reactivated locally in the CNS [38]. Memory CD4^+^ T cells were allowed to migrate for 24 h, after which they were collected for quantification and phenotyping through flow cytometry. The number of migrated cells, calculated as the percentage of cells in the bottom chamber relative to the total number of cells added to the insert or relative to the total number of cells collected after 24 h (top and bottom), displayed significant inter-donor variation. However, no significant effect of tEV treatment was observed when examining the frequency of migrated cells (Fig. S3).

**Fig. 3:**
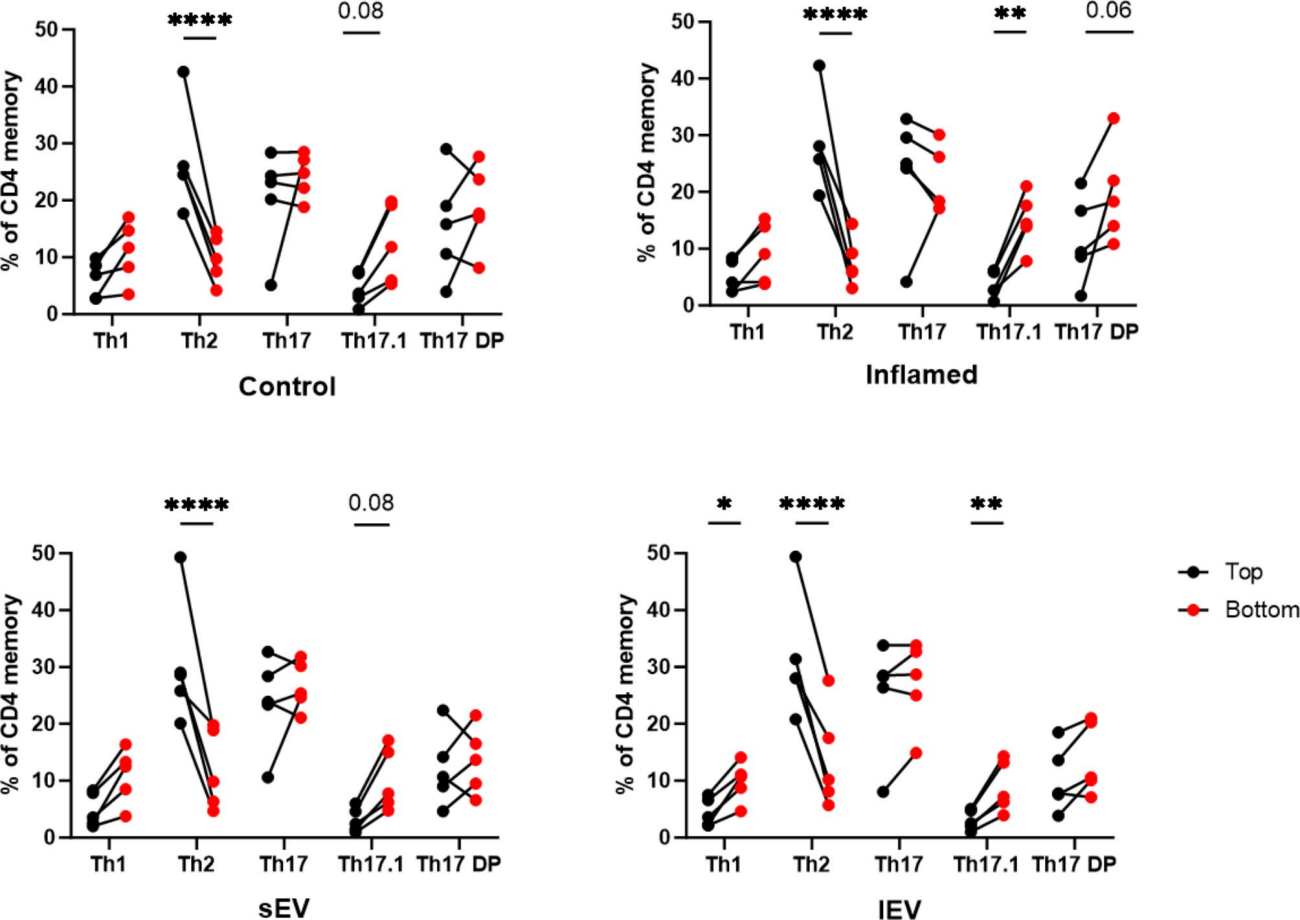
Treatment of BBB-EC with sEV subpopulations creates similar effects to control condition. T cell phenotype depicted as a % of total CD4 memory cells found in the top (black) or bottom (red) compartment in the different conditions. Data is from five different HDs and statistical analysis is done using a two-way ANOVA and Tukey’s multiple comparisons test

A more detailed analysis of the phenotype of migrated cells versus the non-migrated fraction did reveal differences between treatments (Fig. 3). In all conditions, Th2 cells exhibited reduced capacity for migration through the BBB-EC monolayer. In inflammatory conditions, Th17.1 cells, rather than Th1 cells, were significantly enriched in the migrated fraction, while Th17DP cells showed a trend towards enrichment. Treatment of the BBB-EC monolayer with sEV yielded a migration pattern similar to the control condition with only a trend in Th17.1 enrichment. In contrast, lEV treatment induced significant enrichment of both Th17.1 and Th1 cells, suggesting an EV-specific mechanism.

In summary, treatment of BBB-EC with lEV promoted migration of Th17.1 and Th1 cells, whereas sEV treatment did not significantly induce these effects.

### EAE symptoms are exacerbated by lEV and ameliorated by sEV

**Fig. 4:**
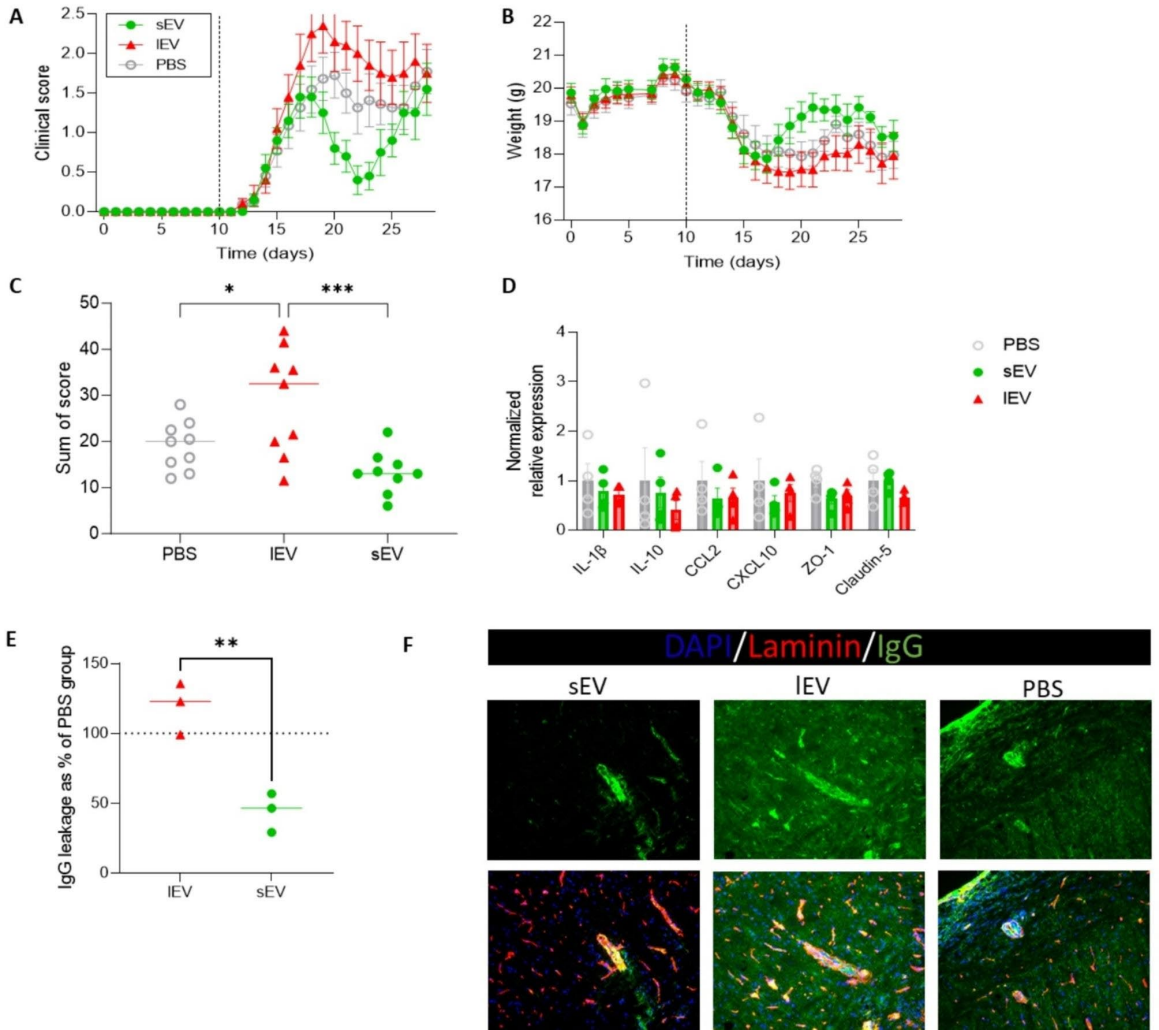
Administration of size-based EV populations affect the EAE disease severity depending on their size. (**A**) Daily clinical scores and (**B**) weights were measures (*n =* 12 per group). Mice were i.v. treated with EV at onset of disease (vertical dotted line). (**C**) Sum of scores were analyzed, leaving out mice with a disease score of 0. (**D**) mRNA expression result on spinal cord collected on day 28 d.p.i. (**E**) Quantification of IgG/ laminin staining in the spinal cord of sEV and lEV injected mice compared to PBS injected mice, set as 100% (*n =* 3–4 / group). (**F**) Representative images showing IgG (green), laminin (red) and DAPI (blue). Statistical analysis was performed using a one-way ANOVA and Tukey’s multiple comparisons test with *p ≤ 0.05, **p ≤ 0.01 and ***p ≤ 0.001 in figures A-D. For figure E statistical analysis was performed using a two-tailed t-test with *p ≤ 0.05, **p ≤ 0.01 and ***p ≤ 0.001 after removal of two outliers using ROUT (Q = 1). Data are depicted as mean ± SEM

Our previous findings have highlighted the size-based effects of BBB-derived extracellular vesicles (BBB-EV) on the migratory capacity of specific Th cell subsets. Given the significant presence of EV in plasma samples of MS patients during relapses [14, 39], we were motivated to investigate the effects of BBB-EV in an in vivo setting. Thus, we administered inflammatory sEV or lEV to mice with EAE at the onset of disease symptoms. Consistent with expectations, mice injected with lEV exhibited exacerbated EAE symptoms compared to mice injected with PBS, as evidenced by the cumulative scores (Fig. 4A–C). Interestingly, analysis of the cumulative scores also revealed a significant difference between lEV- and sEV-injected animals. When examining the disease curve, mice injected with sEV showed a better recovery compared to those injected with lEV or PBS (Fig. 4A). However, after the recovery phase, animals injected with sEV experienced relapses, suggesting a temporary effect of EV in this pathology.

To delve deeper into the functional effects of in vivo EV administration, we measured mRNA expression levels of inflammatory proteins and tight junction molecules (claudin-5 and ZO-1) in the CNS using qPCR. In contrast to our in vitro results, in vivo administration of EV fractions did not result in changes in mRNA levels of the aforementioned proteins (Fig. 4D). Additionally, immunohistochemical staining was performed on the collected spinal cords to quantify the presence of IgG, which serves as an indicator of BBB leakage. This analysis revealed a significant reduction in the area stained with IgG in the sEV-injected group compared to the lEV-injected group (Fig. 4E). Moreover, representative images in Fig. 4F demonstrate better alignment of IgG with laminin in the sEV-treated group, suggesting intact blood vessels within the spinal cord tissue.

To examine the effect of BBB-EV on peripheral immune responses, we performed immunophenotyping on the lymph nodes and spleen at 21 dpi (Fig. S4). This analysis revealed that there were no major changes in the frequencies of immune cell subsets in the spleen, including total T cells (CD3), Th cells (CD4), CTL (CD8), B cells (CD19) and myeloid cells (CD11b). In the inguinal lymph nodes, no statistical significances were identified, although high inter-donor variation was observed. In addition, we examined cytokine production by T cells, and again found no significant changes due to EV injection. Together with the changes in BBB permeability, these data suggest that the effect of BBB-EV on EAE disease scores is mostly derived from central, rather than peripheral effects.

### All inflammatory EV subpopulations increase permeability of the BBB in vitro

**Fig. 5:**
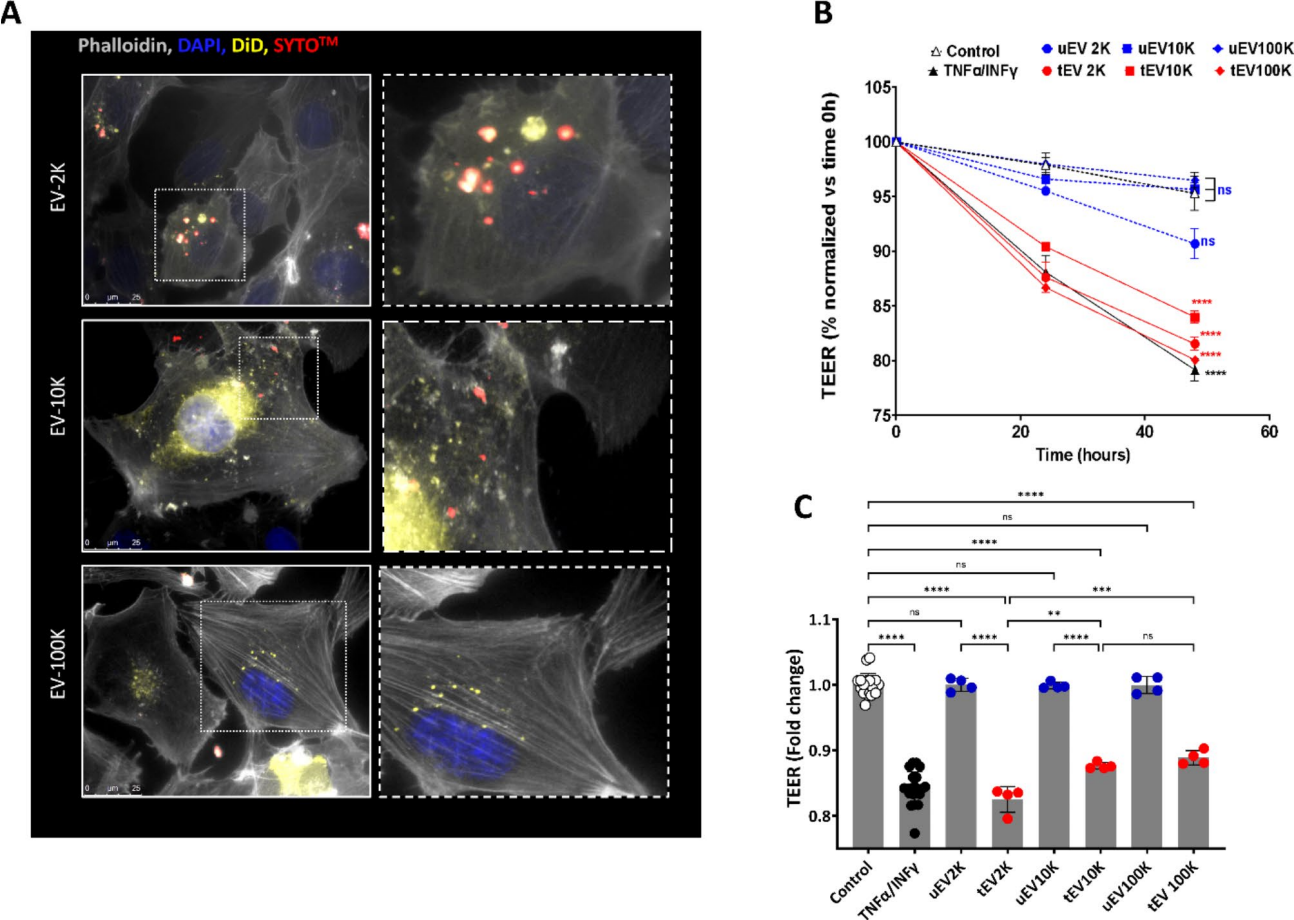
Size-based tEV subpopulations reduce the transendothelial electrical resistance (TEER) of hCMEC/D3 in a dose-dependent fashion. (**A**) In vitro internalization of fluorescently labeled tEV subpopulations (2 K, 10 and 100 K) with DiD and SYTO ^TM^ into hCMEC/D3. (**B**) Real time of TEER measurement of a monolayer of hCMEC/D3 treated with ~ 10^9^ (particle/ml) of tEV and uEV size-based subpopulations in composition with untreated (served as a negative control), TNFα/IFNγ (10 ng/mL) treated (served as a positive control) cells. The percentage of TEER in a monolayer of untreated hCMEC/D3 (served as a negative control) and TNFα/IFNγ (10 ng/mL) (served as a positive control) were compared with different concentrations of different tEV and uEV subpopulations. (**C**) The fold changes in the percentages of TEER values in response to TNFα/IFNγ (10 ng/mL) and different tEV subpopulations (2 K, 10 and 100 K) as normalized to control and their uEV respectively. Data are normalized to time 0 and given as mean ± SEM of at least three independent biological experiments (n ≥ 3) and n = 12 for the control and TNFα/IFNγ groups and one-way analysis of variance with a multiple comparisons test (Dunnet test, p value < 0.05 considered significant) was used to evaluate the statistical significance between treatments versus negative control and Tukey’s test at the value of *p < 0.05 was applied to evaluate the statistical significance between different treatments; *, **, ***, ****: significantly different from controls (p < 0.05, p < 0.01, p < 0.001 and p < 0.0001 respectively)

The contrasting post mortem results on increased leakage found in the CNS but no loss of RNA expression of TJ genes warrants further in vitro studies into the functionality of the BBB-EC. Based on the inflammatory and migration-promoting characteristics identified in BBB-EV we propose that these EV mediate communication between neighboring BBB-EC to propagate BBB disruption. To validate this hypothesis, we first confirmed that BBB-EV are indeed taken up and internalized by resting BBB-EC. Within one hour, EV labeled with membrane marker DiD and nucleic acid marker SyTO were effectively taken up by resting BBB-EC, predominantly accumulating in the perinuclear region (Fig. 5A).

To directly investigate the impact of BBB-EV on the integrity of tight junctions (TJs) and barrier properties to validate the increased BBB leakage found post mortem in EAE, we measured the transendothelial electrical resistance (TEER) of an in vitro human BBB-EC monolayer (hCMEC/D3) as a surrogate marker for BBB tightness. A reduction in TEER indicates increased paracellular permeability and TJ opening. Cells were treated with BBB-EV derived from either resting (uEV) or inflamed BBB-EC (tEV). Following treatment with tEV of all size based EV fractions (which was feasible due to lower amount of EV needed), TEER values decreased to a similar extent as the positive control (TNF-α/IFN-γ) compared to untreated monolayers (Fig. 5B–C, Fig. S6–S7). Treatment with uEV did not affect TEER. Furthermore, the effect of tEV was dose-dependent for all fractions, as demonstrated in Fig. S5. Treatment with a normalized amount of EV (10^9^ particles/ml) revealed that tEV-2 K had a more pronounced effect in reducing TEER values compared to tEV-100 K, confirming their role in compromising BBB-EC integrity (Fig. 5B). To validate that the reduction in TEER values was caused by loss of TJ molecules, a WB was performed on the in vitro human BBB-EC monolayer after 48 h of treatment as described above. The expression of claudin-5 protein was found to be reduced in all conditions when compared to control samples, ZO-1 expression was noticeably lower in tEV2K treated cells. tEV10K treatment only showed a slight reduction while tEV100K remained unchanged (Fig. S7).

In summary, these findings indicate that inflammatory subpopulations of BBB-EV, particularly EV-2 K, are capable of inducing disruption of the BBB and loss of barrier properties.

### BBB-EC respond with different inflammatory responses to size-based EV fractions

**Fig. 6:**
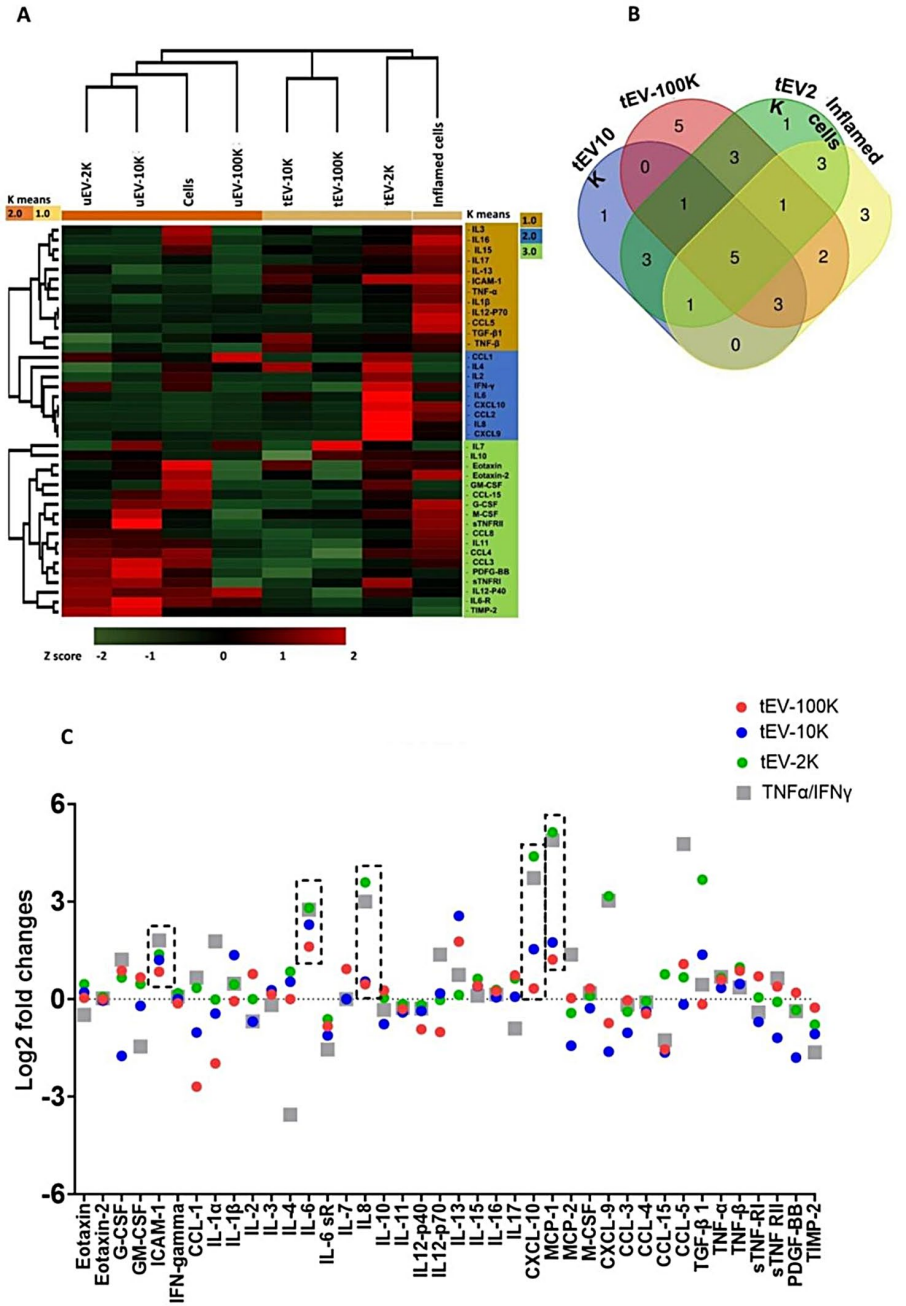
Inflammatory phenotype of hCMEC/D3 in response to the size-based EV subpopulations. (**A**) Heat map and Z-score hierarchical clustering of inflammation-related protein expression (rows) in hCMEC/D3 treated with (~ 10^9^) the size-based tEV subpopulations (2 K,10 and 100 K), TNFα/IFNγ treated cells and untreated cells. Clustering in rows and columns was done based on the Euclidean distance metric of 2–3 K-mean Value and a complete linkage method. The colors of the heat map indicate the highly expressed (red) and the basal expression levels (green) based on their Z-score. Euclidean distance also represents the close neighbors to the selected column (TNF-α– treated cells) according to their overall similarity. (**B**) Venn diagrams display the overlap of the expressed inflammatory proteins in hCMEC/D3 treated with (~ 10^9^) of size- based tEV subpopulations (2 K,10 and 100 K) and TNFα/IFNγ treated cells. (**C**) Differential expression of inflammation-related proteins (log2 (fold change vs. ctrl)) in cells following tEV (~ 10^9^) and inflammation stimulation

To gain further insights into the inflammatory cascade triggered in BBB-EC treated with different EV subpopulations, we profiled the inflammatory protein content of BBB-EC using antibody arrays after treatment with a normalized amount of each EV subpopulation. The results were analyzed using clustered heatmaps and Venn diagrams (Fig. 6A–B). We identified two distinct clusters with differences in their inflammatory responses. Specifically, cells treated with tEV-2 K and inflammatory cells clustered together, indicating that the tEV-2 K fraction induced a similar pattern of inflammation in BBB-EC as the positive control (TNF-α/IFN-γ). A total of 32 inflammation-related proteins showed differential expression across all treatments. Among them, 5 out of 32 proteins (15.6%) including CCL2, CXCL10, IL-6, IL-8, and ICAM-1 were highly expressed in BBB-EC in response to different tEV fractions and TNF-α/IFN-γ (Fig. 6A and Table S2). Additionally, IL-1β, a hallmark of inflammation, was exclusively expressed in cells treated with either tEV-2 K and tEV-10 K or TNF-α/IFN-γ. Interestingly, lEV (EV-2 K and EV-10 K) and sEV (EV-100 K) induced distinct inflammatory cascades in BBB-EC (Fig. 6C).

**Fig. 7:**
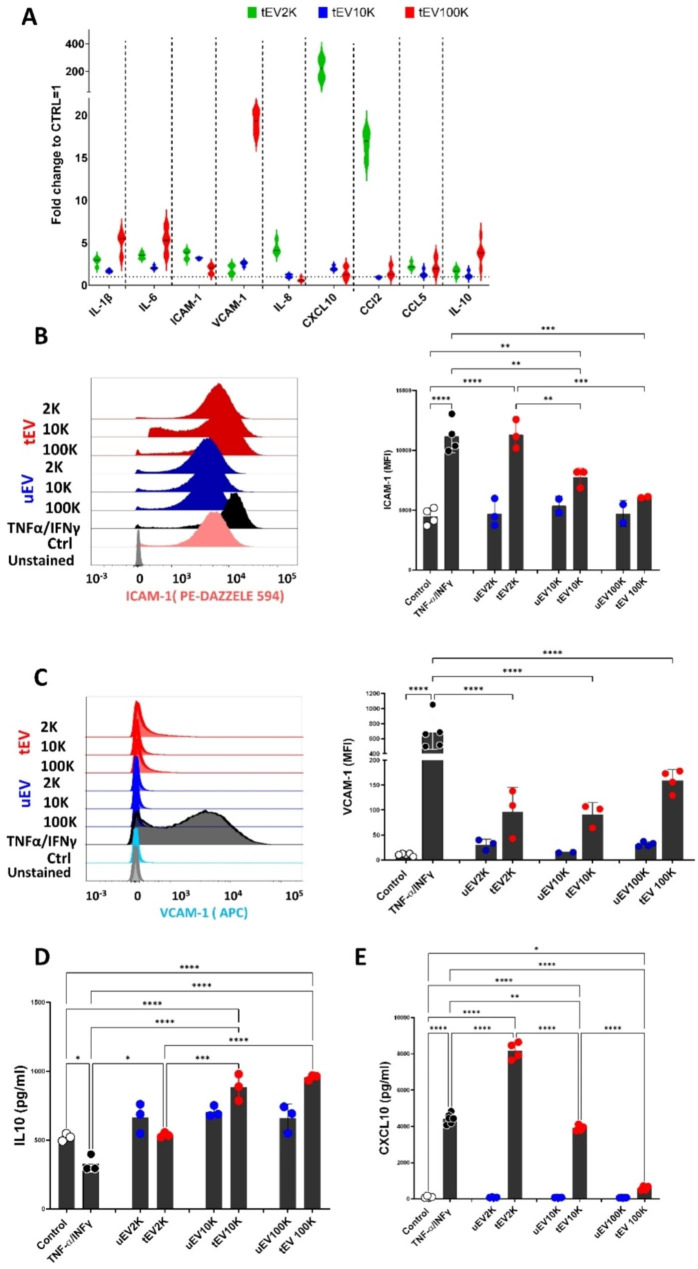
EV subpopulations increase the production of key pro-inflammatory markers in hCMEC/D3 cells. (**A**) mRNA expression of inflammatory candidate genes (IL6, IL1β, ICAM-1 and VCAM-1) in hCMEC/D3 treated with (normalized to 10^9^) of the size-based tEV subpopulations (2 K,10 and 100 K), TNFα/IFNγ treated cells and untreated cells. Data were analyzed using two stable reference genes and the fold changes were plotted as the mean ± SEM and normalized to non-stimulated control cells (n = 3 biological replicates per group). (**B**–**C**) Representative flow cytometry data (left) and quantification (right) of ICAM-1 (**B**) and VCAM (**C**) level hCMEC/D3 in response to size-based EV subpopulations (2 K,10 and 100 K) and in comparison with TNFα/IFNγ (10 ng/mL) and untreated hCMEC/D3 as positive and negative controls, respectively. (**D**–**E**) Protein levels from IL-10 (**D**) and CXCL10 (**E**) produced by hCMEC/D3 in response to size-based EV subpopulations (2 K,10 and 100 K) and in comparison, to TNFα/IFNγ (10 ng/mL) and untreated hCMEC/D3 as positive and negative controls, respectively. Data are presented as mean ± SEM (*p < 0.05)

Based on these results, we observed high expression of IL-1β, CCL-2, CCL-5, CXCL-10, IL-6, IL-8, and IL-10 in BBB-EC treated with both tEV. Adhesion molecules such as ICAM-1 and VCAM-1, known to play significant roles in T cell migration and junctional reorganization, are regulated by inflammation. Therefore, we quantitatively validated the expression of these effector molecules at the protein and mRNA levels in EV-treated BBB-EC (Fig. 7A–E). mRNA expression analysis by qPCR revealed distinct patterns of inflammatory and adhesion molecules in untreated and EV-2 K and EV-10 K-treated cells compared to EV-100 K (Fig. 7A). ICAM-1 was found differentially expressed in both lEV fractions (tEV-2 K and tEV-10 K) while CCL2, CXCL-10, and IL-8 were exclusively differentially expressed in tEV-2 K treated cells. Further, increased expression of IL-10 and VCAM-1 was only detected in sEV (tEV-100 K) -treated cells (Fig. 7A). Consistent with qPCR results, flow cytometric analysis revealed higher levels of ICAM-1 protein in cells treated with tEV-2 K and tEV-10 K but not tEV-100 K when compared to control cells. When compared to inflamed BBB-EC tEV-10 K tEV-100 K showed significant lower levels of ICAM-1 protein. Surprisingly, no significant differences were found for VCAM-1 protein between the tEV treated BBB-EC and control cells while lower VCAM-1 protein levels were found in in all EV-treated cells when compared to inflamed EC (Fig. 7C). Flow cytometric analysis of VE-Cadherin levels showed no differences (Fig. S8). Finally, the expression levels of CXCL10 and IL-10 proteins were quantitatively verified using ELISA on the supernatants of EV-treated BBB-EC. This confirmed that cells treated with tEV-100 K exhibited higher levels of IL-10 production than tEV-2 K and inflamed cells but in contrast to mRNA expression tEV-10 K treated cells also showed increased IL-10 production compared to tEV-2 K, control and inflamed BBB-EC (Fig. 7D). Consistent with the qPCR results, CXCL10 was significantly higher in tEV-2 and 10 K -treated BBB-EC compared to tEV-100 K treated EC (Fig. 7E).

Overall, these findings demonstrate that the different size-based fractions derived from inflammatory BBB-EC elicit different responses in their target cells. Noteworthy, tEV-10 K mediated effects appear to be intermediate related to tEV-2 K and tEV-100 K mediated effects.

## Discussion

Recently, increasing evidence has shown the involvement of EV in the development of MS. However, studies about their role in vivo as well as the effect of different size-based EV on the BBB functioning were missing. Therefore, we aimed to gain a more profound understanding of the impact of BBB-EC derived EV on the functional properties of the BBB and on the disease progression of EAE induced mice. This study characterizes different sized-based EV fractions as well as their impact in vitro as well as in vivo.

Most studies in the field of EV research apply differential centrifugation for the enrichment of large-sized EV, and small-sized EV fractions derived from different cell types at g-forces of 10,000–20,000 x g (10-20 K) and > 100,000 x g (100 K), respectively [16, 40]. In this study these three different fractions of EV were isolated and characterized following MISEV2018 guidelines. In order to avoid presence of apoptotic bodies in the lEV fraction (as indicated by the MISEV guidelines), BBB-EC were only grown up to 80–90% confluency to minimize apoptosis. Patches of confluent EC layers were always present, however we cannot rule out that EV derived from 100% confluent layers would have resulted in a different outcome. This is a limitation of the study, because our results might not fully reflect the in vivo EV production by BBB-EV, and should be considered in further studies.

Based on the NTA results, a 3- to 4-fold increase in EV production was revealed in hCMEC/D3 cells after inducing inflammation to these cells. This is in line with previous reports on EV production after TNF-α stimuli [40]. Further, an overlap in mean size and number of EV produced by the hCMEC/D3 cells in the EV-2 and 10 K fractions suggests that hCMEC/D3 cells release only two major size-based populations of EV. This finding however, is not unexpected as one of the drawbacks of differential centrifugation is the inability to separate exosomes from microvesicles completely. Based on these results it can be concluded that inflamed BBB-EC produce only two distinct size-based EV populations, namely sEV (EV-100 K) and lEV (EV-2 K and EV-10 K). Indeed, content and pathway analysis of these size-based EV fractions released by the BBB-EC support the finding of only two distinct EV fractions.

Increased T cell migration is a major player in MS pathogenesis. Here, we revealed that treatment of BBB-EC with lEV resulted in a significantly higher percentage of Th1 cells in the migrated fraction, and this effect was not seen in the other conditions. This effect is possibly mediated by the increased presence of CXCL10 protein as this is considered a Th1 associated cytokine capable of coordinating Th1 migration [41]. Interestingly, Th1 cells have only been found significantly increased in the CNS of MS patients after clinical development of the disease. This is in contrast with the infiltration of Th17 cells which occurs even before clinical symptoms in mice [42]. In this study, we found no differences in Th17 cell migration after treatment of the BBB-EC layer, except when looking at the Th17.1 subset. This Th1-like Th17 subset was found significantly higher in all migrated fractions except for the sEV condition. The Th17.1 subset is found to have a dominant contribution to the MS disease and are known to preferentially migrate into the CNS [43]. Moreover, patients treated with natalizumab showed a decrease in Th17.1 in the CSF and an increase within the peripheral blood, indicating their importance in disease pathogenesis [44]. In the current study, content analysis of BBB-EV remained limited to protein analysis, while a full analysis of the content including miRNA could shed light on other potential mechanisms by which this effect on T cell migration is mediated.

It is known that in physiological conditions, EC are exposed to circulating EV in the bloodstream. Therefore, it seems likely that these EC are responsive to EV-mediated signaling or are able to engulf these EV. Our data show that BBB-EC are indeed capable of taking up BBB-EV in vitro. To elucidate the true biological effect of the different BBB-EV subsets, we conducted an in vivo study where EAE was induced and size-based EV fractions were administered on day of onset. When looking at disease progression a clear difference is seen between the different fractions where sEV show a milder EAE course. This difference can possibly be attributed to the increased IL-10 production by sEV treated BBB-EC found in vitro, as previous research showed that IL-10 treatment during EAE also weakened the disease course [45]. It is also known that IL-10 production by monocytes inhibits the Th17 migration into the CNS in EAE and therefore attenuates the disease course [46]. The difference in disease score could also be explained by less BBB leakage as shown by post mortem staining of CNS tissue for IgG and laminin. However, post mortem analysis of EAE tissue showed no difference in gene expression of tight junction molecules. To rule out effects on peripheral immune responses, cells obtained from the spleen and lymph nodes of EV-treated EAE mice were analyzed and no difference was found in the lymphocyte composition or their production of inflammatory cytokines in spleen-derived cells. These data suggest that the effects on EAE disease course are not mediated by peripheral immune effects, and are at least in part caused by effects on the BBB. Finally, although human derived EV were injected, these data combined with a reduction in EAE score in the sEV group, highlight that there is no xenogeneic immune response. This is in line with a previous report, where EV derived from a human cell line were also injected into mice, and did not elicit a xenogeneic immune response [47]. Of note, high inter-donor variation was observed in lymph node-derived cells, which warrants further investigation.

Interestingly, we found a significant downregulation of barrier integrity when BBB-EC were exposed to EV, and that tEV-2 K had a greater disruptive impact on the integrity when compared to sEV exposure. This result is in line with the content analysis where we identified that tEV-2 K showed the biggest overlap with inflamed BBB-EC. Besides the effect on functionality of the BBB, we also found a phenotypical change of BBB-EC after exposure to EV. In general, the phenotype showed a switch to a more inflammatory state after EV exposure, although there were some size-based differences. sEV resulted in increased expression of the cytokine IL-10, which is capable of down-regulating the synthesis of pro-inflammatory cytokines [48]. This would indicate a more protective phenotype of the size-based sEV fraction. In line with this, chemokines CCL2 and CXCL-10 and the pro-inflammatory cytokine IL-8 were found exclusively in the EC cells exposed to the EV-2 K fraction. This is in accordance with other reports showing reduced mRNA levels of IL-10 in MS patients when compared to HDs as well as lower levels in serum [49, 50]. In addition, IL-10 levels were rescued in MS patients treated with IFN-β-1b [51], suggesting a protective role for IFN-β-1b treatment on BBB integrity. Notably, a protein array was used to assess the content of EV, with the limitation that not the entire proteome was identified in these EV fractions. In addition, EV are known to contain non-protein components as well (e.g. miRNA). Therefore, differences in size-based fractions cannot be attributed to a single factor but rather to a combination of factors contained within a certain size-based fraction.

Overall, our findings highlight the active involvement of BBB-EV in the perpetuation of EC inflammation and the disruption of the BBB, both in vitro and in vivo. Although effects of EV on the BBB integrity and disease score seen in vivo could possibly be mediated by indirect effects, the similarity between our in vitro and in vivo data indicate a clear effect on BBB-EC. The differential effects of size-based EV fractions suggest their dual role in the pathogenesis of MS, and warrants future research into this topic. However, results found in this manuscript are related to hCMEC/D3-derived EV, and validation in primary cells and/or iPSC-derived cells is preferable. Still, our study contributes to the growing understanding of EV-mediated communication in neuroinflammation and opens avenues for the development of novel therapeutic and diagnostic strategies for MS.

### Electronic supplementary material

Below is the link to the electronic supplementary material.

Supplementary Material 1: Supplementary Figures and Tables

## Data Availability

The datasets used and/or analysed during the current study are available from the corresponding author on reasonable request.

## Abbreviations

MS: Multiple sclerosis
BBB: Blood-brain barrier
BBB-EC: Blood brain barrier endothelial cell
TJs: Tight junctions
AJs: Adherence junctions
EV: Extracellular vesicles
sEV: Small extracellular vesicles
lEV: Large extracellular vesicles
