# Supplementary material for: Cerebral microvascular endothelial cell-derived extracellular vesicles regulate blood − brain barrier function
Source: Fluids Barriers CNS. 2023 Dec 19;20:95. doi: 10.1186/s12987-023-00504-6 (PMC10729529; doi:10.1186/s12987-023-00504-6)
Supplement: Supplementary file 1 — Supplementary Material 1: Supplementary Figures and Tables [file 12987_2023_504_MOESM1_ESM.docx]

**Cerebral microvascular endothelial cell-derived extracellular vesicles contribute to blood−brain barrier disruption**

Hosseinkhani Baharak ^1,2,3^*^#^, Duran Gayel^1,2#^, Hoeks Cindy^1,2^, Hermans Doryssa^1,2^, Schepers Melissa^1,2,4,5^ Baeten Paulien^1,2^, Poelmans Joren^1,2^, Coenen Britt^1,2^, Bekar Kübra^1,2^, Pintelon Isabel^6^, Timmermans Jean-Pierre^6^, Vanmierlo Tim^1,2,4,5^, Michiels Luc^7^, Hellings Niels^1,2^, Broux Bieke^1,2^*

^1^University MS Center, Campus Diepenbeek, Diepenbeek, Belgium

^2^Neuro-Immune Connections and Repair Lab, Department of Immunology and Infection, Biomedical Research Institute, UHasselt, Diepenbeek, Belgium

^3^Laboratory of Angiogenesis and Vascular Metabolism, Center for Cancer Biology (CCB), VIB and Department of Oncology, Leuven Cancer Institute (LKI), KU Leuven, Leuven, Belgium

^4^ Department Psychiatry and Neuropsychology, School for Mental Health and Neuroscience, Maastricht University, Maastricht, The Netherlands.

^5^ Department of Neuroscience, Biomedical Research Institute, Faculty of Medicine and Life Sciences, Hasselt University, Hasselt, Belgium.

^6^Laboratory of Cell Biology & Histology / Antwerp Centre for Advanced Microscopy (ACAM), University of Antwerp, Universiteitsplein 1, Antwerp 2610, Belgium

^7^Bionanotechnology group, Biomedical Research Institute, UHasselt, Diepenbeek, Belgium

* These authors share senior authorship

#These authors share first- authorship

*** To whom correspondence may be addressed:** prof. Dr. Bieke Broux, Universiteit Hasselt- Campus Hasselt, Martelarenlaan 42- B-3500 Hasselt, T +32(0)11 26 93 13, bieke.broux@uhasselt.be


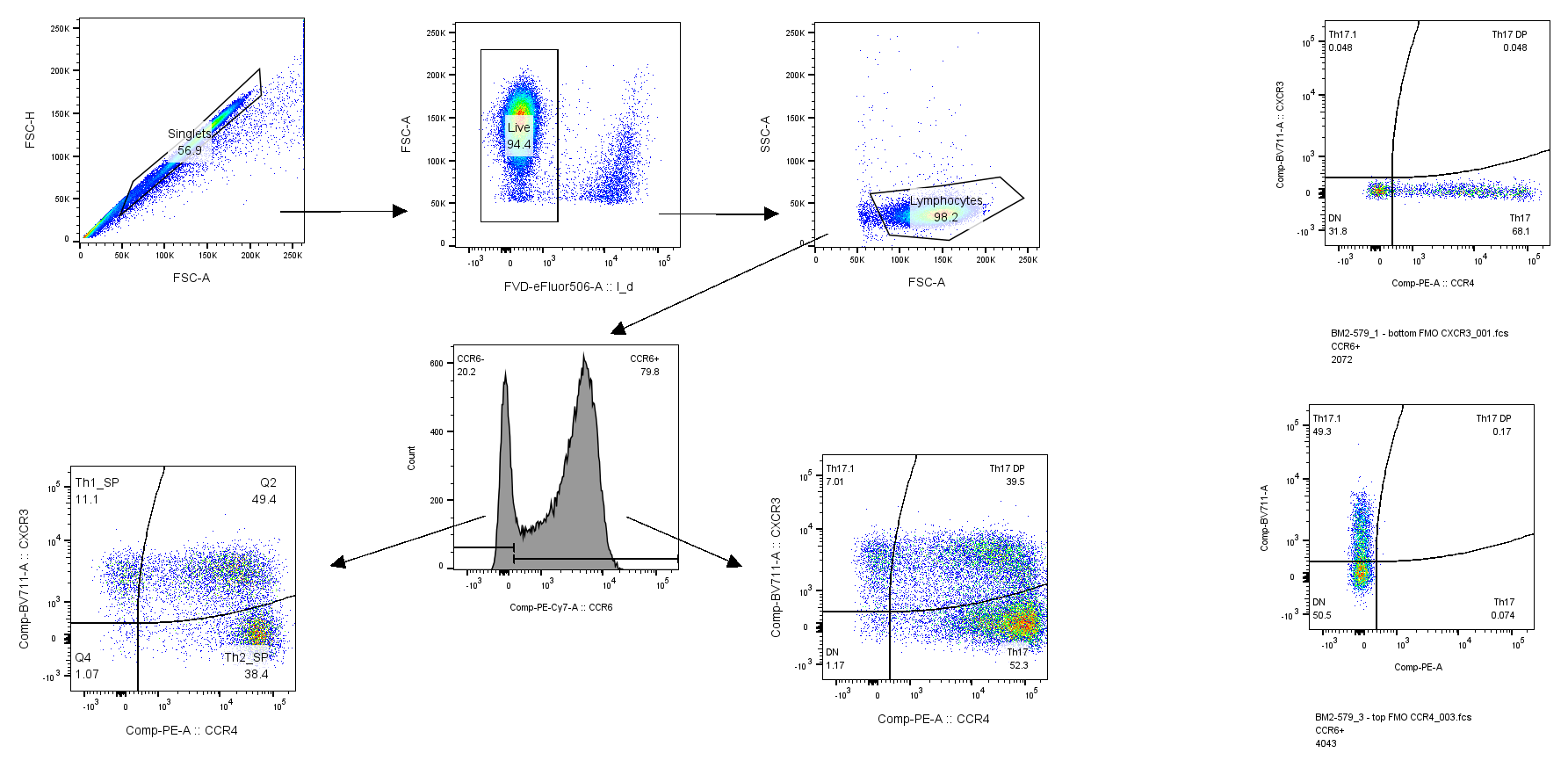


**Figure S1:** Gating strategy for flowcytometric analysis on migrated and non-migrated CD4 memory T cells over an inflamed or EV treated hCMEC/d3 coated insert. Gating is based on fluorescence minus one principle gates.

**
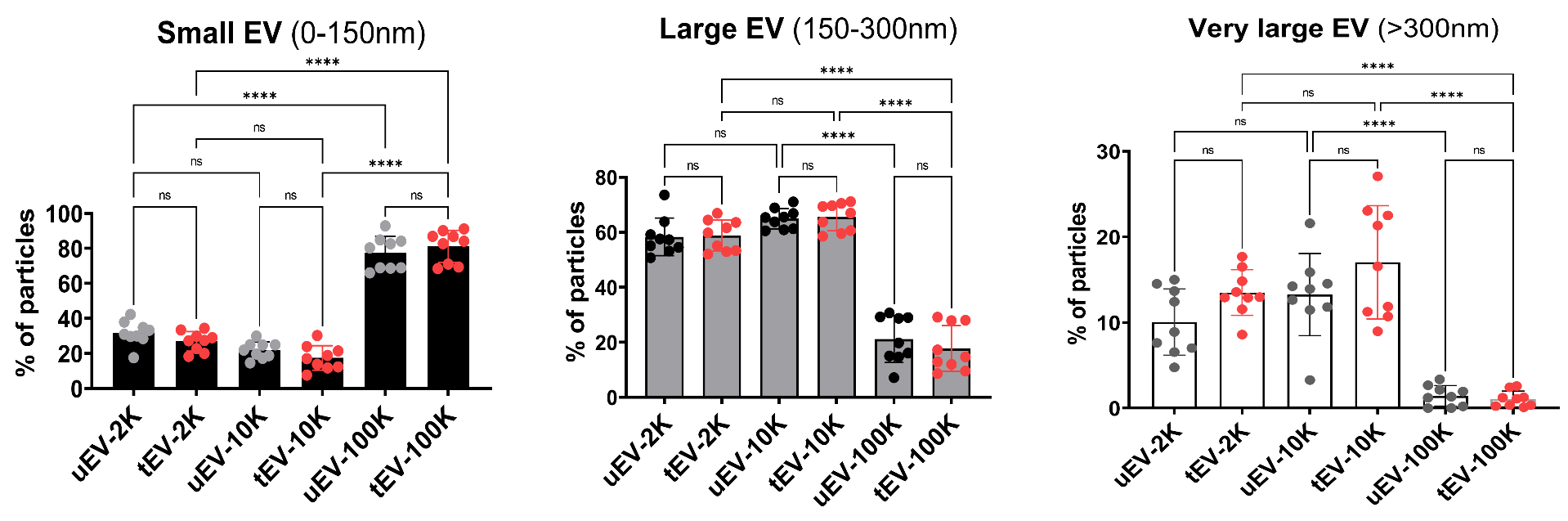
**

**Figure S2A:** The percentage of small EV (0- 150 nm), large EV (150- 300 nm) and very large EV (>300 nm) in different size-based subpopulations of EV derived either from untreated cells (uEV; gray dots) or TNFα/IFNγ inflammatory triggered (tEV; red dots) cells. Inflammation does not impact on size profile of EV released by BBB-EC (uEV vs tEV). The EV-10 K subsets predominantly contained of small-sized ((81.1±9.1%), while the two fractions of 2K and 10K were dominated by large- (50–100 nm) and very large-sized EV (with a distribution of 65.5±5.1% and 58.8±5.6% respectively). There was no significant difference in the percentage of small EV, large EV and very large EV (>300 nm) of EV2K and 10K subpopulations. Data are presented as means ± SD (n ≥ 6 independent biological experiments, 1 symbol per batch) and one-way ANOVA Tukey's multiple comparison was used to determine significance between multiple groups: ns, no significance, *p < 0.05 **P < 0.01, ****P < 0.0001.

**
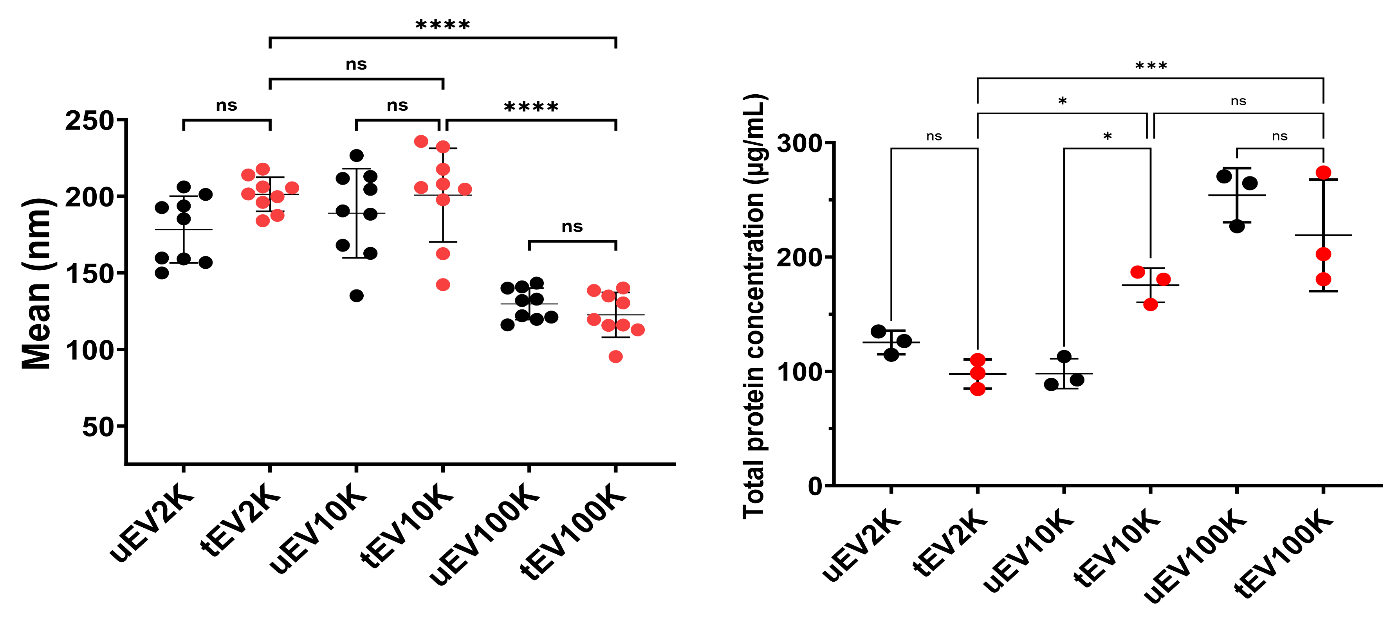
**

**Figure S2B:** Mean size (nm) and total protein concentration (µg/ml) in different size-based subpopulations of EV derived either from untreated cells (uEV; black dots) or TNFα/IFNγ inflammatory triggered (tEV; red dots) cells.

**Table S1:** Grouped inflammatory proteins between EV size- based populations (EV-2K, EV-10K and EV-100K )and TNFα/IFNγ treated hCMEC/D3in Venn diagram produced using open source software (<http://bioinformatics.psb.ugent.be>).

| **Conditions** | **Total** | **Elements** |
| --- | --- | --- |
| EV-2K, EV-10K, EV100K and TNFα/IFNγ treated hCMEC/D3 | 6 | CCL2, CCL5, IL6, IL-8, ICAM-1, CXCL10 |
| EV-2K, EV-10K, EV100K | 2 | CCL8, CXCL9 |
| TNFα/IFNγ treated hCMEC/D3 | 2 | IL15 and IL16 |
| EV-2K, , | 1 | IL1-β |
| EV100K | 3 | CCL1, IL10 and IL17 |


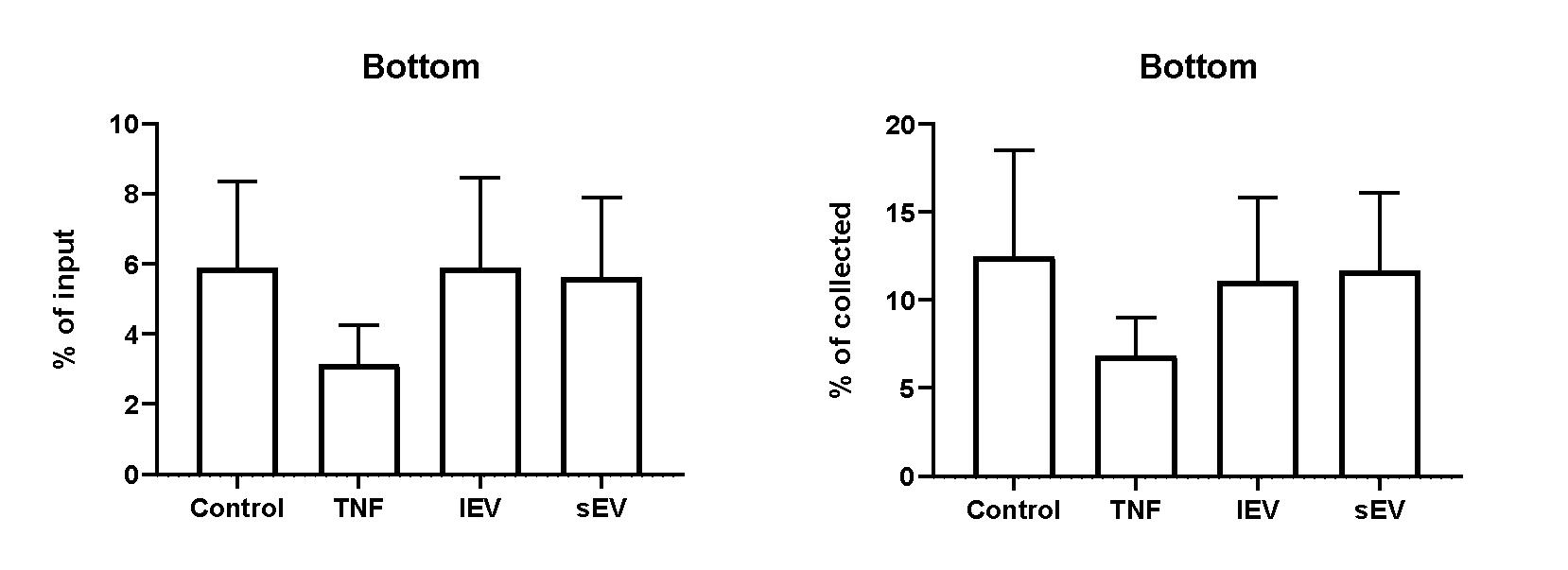


**Figure S3:** Left: percentage of cells found in bottom compartment after 24 h as a percentage of total plated CD4^+^ memory T cells in different conditions of BBB-EC treatment. Right: percentage of cells found in bottom compartment after 24 h as a percentage of total CD4^+^ memory T cells found (top + bottom) in different conditions of BBB-EC treatment. Data is from five different HDs and statistical analysis is done using a one-way ANOVA and Tukey’s multiple comparisons test. No statistical differences were found.

**Figure S4:** Peripheral immune cell phenotypes after i.v. EV administration in MOG immunized mice. No significant changes were observed on all immune cell subsets as well as the production of inflammatory cytokines by CD3+ cells.


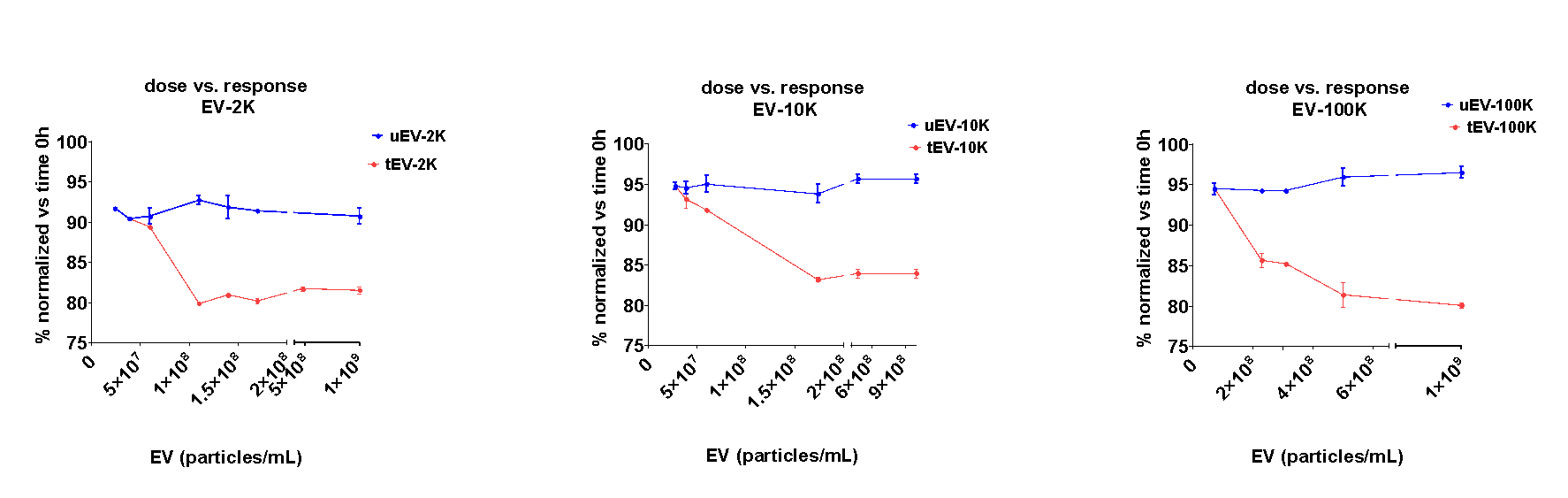
**Figure S5:** Dose-dependent reduction in TEER values of hCMEC/D3 cells (% normalized to 0h time) after treatment with size-based EV fractions for 48h.

**
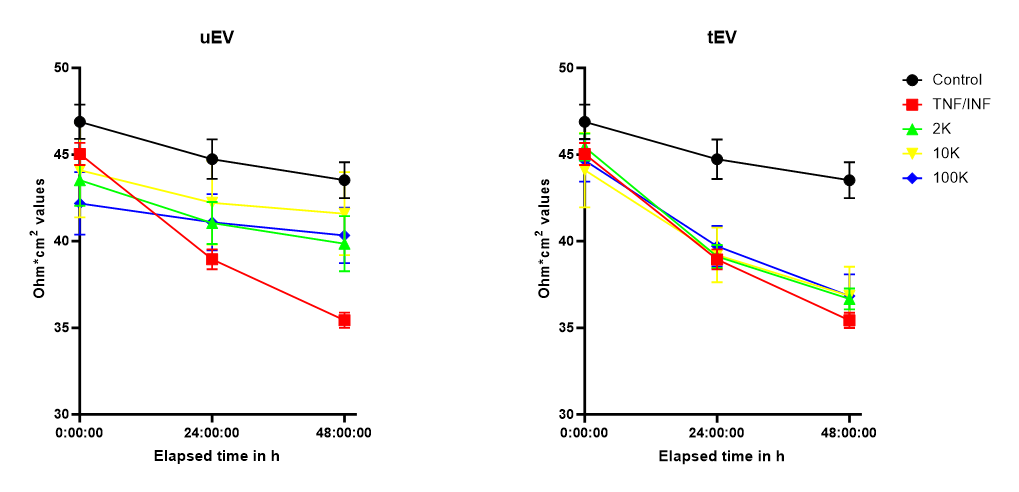
**

**Figure S6:** Absolute TEER values without normalizing to time point or control condition elapsed over 48h. Data is represented as mean ± SEM.

**
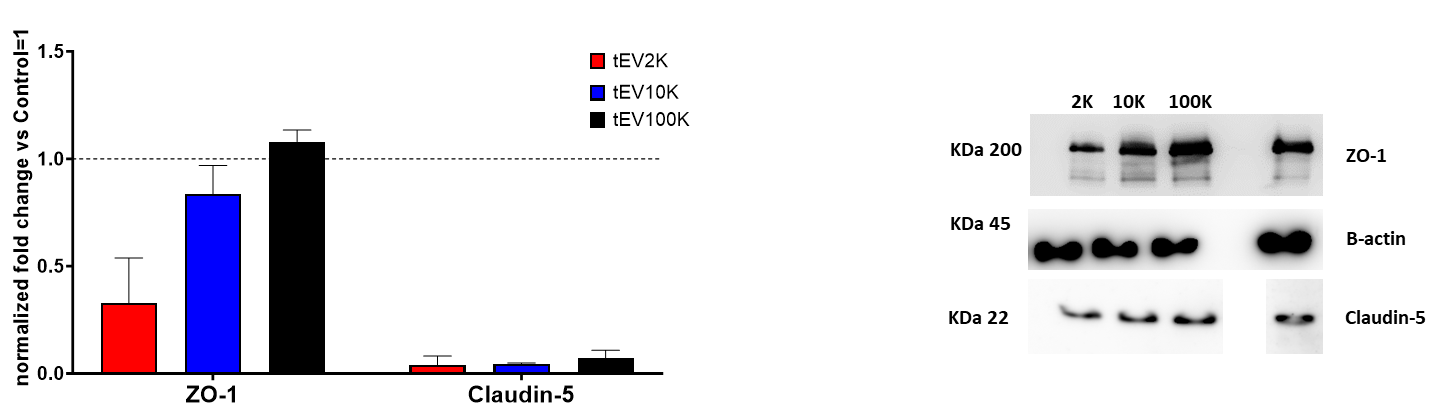
**

**Figure S7**: Representative western blot showing ZO-1 (200 kDa) and Claudin-5 (22kDa) protein expression in untreated hCMEC/d3 or with tEV size-based EV subpopulations (concentration normalized to 1E09). Relative intensity of target proteins to β-actin was calculated. Data are represented as mean ± SEM of two independent experiments (n= 2).


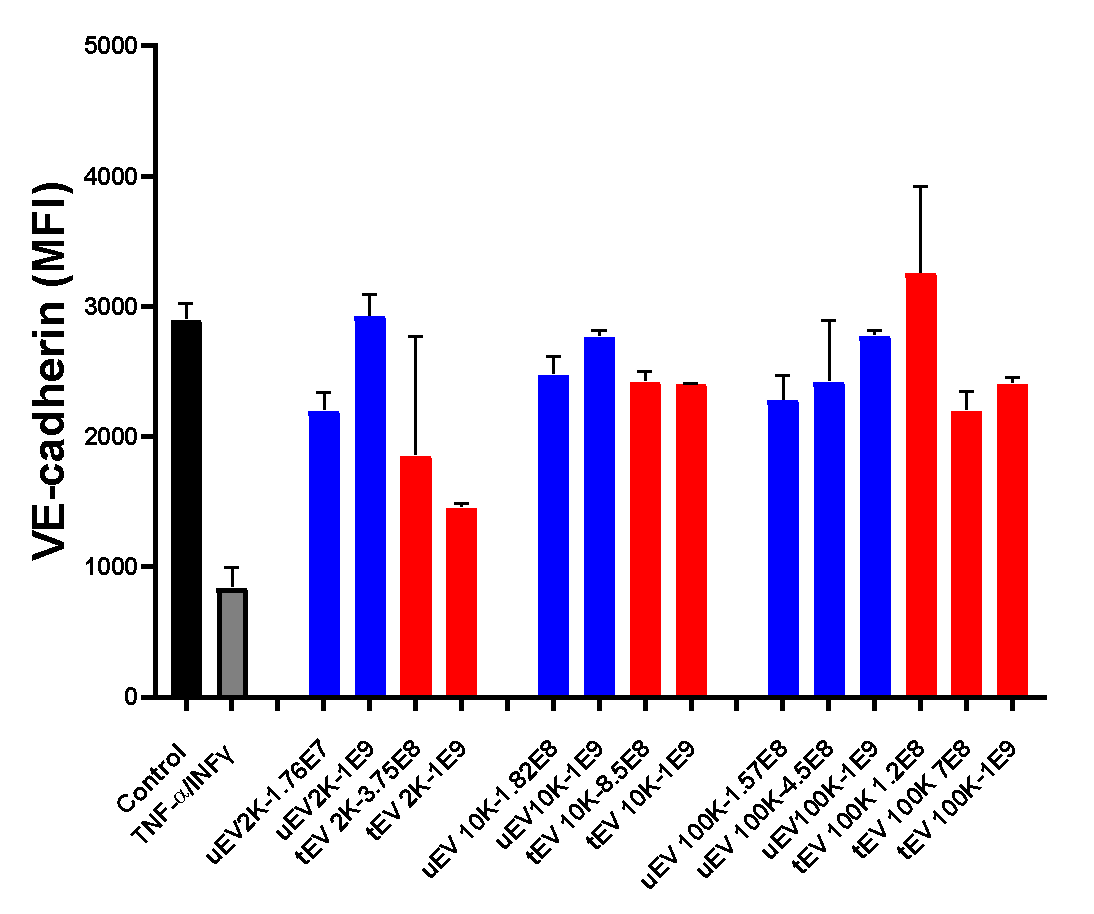


**Figure S8:** VE-cadherin level hCMEC/D3 in the response to increasing doses of size- based EV subpopulations (2K,10K and 100K) and in comparison, to TNFα/IFNγ (10 ng/mL) and untreated hCMEC/D3 as positive and negative controls, respectively. One-way analysis of variance with a multiple comparisons test (Dunnet test, p-value < 0.05 considered significant) was used to evaluate the statistical significance between treatment versus negative control (<0.0001), and Tukey’s test at the value of *p < 0.05 () was applied to evaluate the statistical significance between different treatments; *, **, ***, ****: significantly different from controls (p < 0.05, p < 0.01, p < 0.001 and p < 0.0001, respectively).

**Table 3:** Grouped inflammatory proteins between cells-treated with different EV size- based populations (EV-2K, EV-10K and EV-100K )and TNFα/IFNγ treated hCMEC/D3in Venn diagram produced using open source software (<http://bioinformatics.psb.ugent.be>).

| **Conditions** | **Total** | **Elements** |
| --- | --- | --- |
| EV-2K, EV-10K, EV100K and TNFα/IFNγ treated hCMEC/D3 | 5 | CCL2, IL8, IL6, ICAM-1, CXCL10 |
| EV-10K, EV100K and TNFα/IFNγ treated hCMEC/D3 | 3 | IL13, TNF-α and TNF-β |
| EV-2K, EV-10K, EV100K | 1 | IL15 |
| EV-2K, EV-10K, and TNFα/IFNγ treated hCMEC/D3 | 1 | IL1- β |
| EV-2K, EV-100K, and TNFα/IFNγ treated hCMEC/D3 | 1 | CSF 3 |
| EV-2K, EV-10K, | 3 | IFN-γ, IL3, CCL11 |
| EV-2K, EV100K | 3 | IL16, IL17 and CSF2 |
| TNFα/IFNγ treated hCMEC/D3 and EV100K | 2 | CCL-5, CD120B |
| TNFα/IFNγ treated hCMEC/D3 and EV-2K, | 2 | CCL1, TGF- β, CXCL9 |
| EV-100K | 5 | MCSF, IL2, IL7, IL10 and CD120A |
| EV-2K | 1 | INF-γ |
| TNFα/IFNγ treated hCMEC/D3 | 3 | IL12, CCL8, IL1-α |
